# Community-based rehabilitation services implemented by multidisciplinary teams among adults with stroke: a scoping review with a focus on Chinese experience

**DOI:** 10.1186/s12889-024-18218-1

**Published:** 2024-03-07

**Authors:** Zifen An, Ke Li, Xinyi Yang, Jie Ke, Yuying Xu, Xi Zhang, Xianmei Meng, Xianwu Luo, Liping Yu

**Affiliations:** 1 School of Nursing, Wuhan University, No. 115 Donghu Road, 430071 Wuhan, Hubei Province China; 2 Department of General Practice, Zhongnan Hospital of Wuhan University, No. 169 Donghu Road, 430071 Wuhan, Hubei Province China

**Keywords:** Community-based rehabilitation, Multidisciplinary, Adults, Stroke, Scoping review

## Abstract

**Background:**

Despite the growing interest in hospital rehabilitation services for communities, studies on existing community-based rehabilitation (CBR) services remain scarce owing to limitations in the development of community health services and regional cultural diversity. As a guaranteed measure for ensuring the quality of rehabilitation services and achieving the desired service outcomes, clear roles and responsibilities in multidisciplinary teams and effective service delivery are particularly important.

**Objective:**

This scoping review aimed to determine the scope of community stroke rehabilitation programs involving existing multidisciplinary teams and to analyze the implementation content and implementers’ functional roles to provide guidance for future CBR programs.

**Methods:**

The scoping review design followed the methodology of the Joanna Briggs Institute and was based on the normative scoping review framework proposed by Arksey and O’Malley. The comprehensive CBR framework was proposed by World Health Organization-guided data charting and analysis.

**Results:**

Of the 22,849 identified citations, 74 studies were included, consisting of 6,809 patients with stroke and 49 primary caregivers, most of whom were from China. The most common working mode in CBR programs was a dual approach involving both healthcare professionals in medical institutions and community healthcare professionals. The number of programs in each discipline was in the following descending order: nursing, medical care, rehabilitation, psychology, nutrition, and public health. Among these, multidisciplinary teams comprising medical, nursing, and rehabilitation disciplines were the most common, with a total of 29 programs. Disciplinary members were mainly responsible for implementing their respective disciplinary content, with physicians providing guidance for the programs. More than 82.4% of the studies reported 2–4 intervention strategies. The intervention forms of rehabilitation content were the most diverse, whereas preventive interventions were more homogeneous than others. Physical function and socio-psychological measurements were the most commonly reported outcomes.

**Conclusion:**

CBR services implemented by multidisciplinary teams can effectively achieve functional and emotional improvement in patients with stroke, and nurses are the most involved in implementation, especially in community settings. The results further emphasize the importance of strengthening the exploration of nurses’ maximum potential to implement CBR plans in future practice.

**Trial registration:**

The registration information for this scoping review can be found at osf.io/pv7tg.

**Supplementary Information:**

The online version contains supplementary material available at 10.1186/s12889-024-18218-1.

## Introduction

Stroke is a leading cause of morbidity, mortality, and disability around the world [1]. In 2019, there were 12.2 million incident strokes and 101 million prevalent strokes globally, representing a 70% increase compared with new stroke statistics for 1990 [1]. The number of new stroke cases in China was as high as 3.94 million in 2019, and the incidence of stroke increased by 86% compared with that in 1990 [2]. Although the mortality rate of stroke has been decreasing generally, the disability rate of post-stroke patients remains high owing to residual cognitive, language, physical, and other functional disorders [1, 3]. Approximately 143 million post-stroke patients were disabled and required continuous long-term supportive services in 2019, resulting in heavy physical, psychological, and financial burdens on patients and their families [1]. In addition, according to the World Stroke Organization, the annual global cost related to stroke was as high as US $721 billion (accounting for 0.66% of global gross domestic product (GDP)), and the clinical and economic burden caused by stroke was of great significance to public health [4].

Rehabilitation services are key to promoting functional recovery, relearning skills, and achieving independence in patients with stroke [5]. Although many patients undergo multiple rehabilitation stages or move back and forth between different care settings to achieve their rehabilitation goals, most stroke survivors eventually return to their families to receive community-based rehabilitation (CBR) services [6]. CBR, first launched by the World Health Organization (WHO) following the International Conference on Primary Health Care (the Almaty Declaration) in 1978, is a strategy to improve access to rehabilitation services for persons with disabilities in developing countries [7]. In 1981, the WHO Expert Committee on Disability Prevention and Rehabilitation defined CBR as follows: ‘Community-based rehabilitation involves measures taken at the community level to use and build on the resources of the community, including the impaired, disabled, and handicapped persons themselves, their families, and their community as a whole’ [8]. Subsequently, the concept of CBR has expanded continuously. In 2004, the International Labor Organization, the United Nations Educational Scientific and Cultural Organization, and the WHO redefined CBR as a strategy within general community development for rehabilitation, poverty reduction, equalization of opportunities, and social inclusion of all people with disabilities [9]. However, although this concept has been well defined, CBR programs for patients with stroke still lack several key components.

**Fig. 1:**
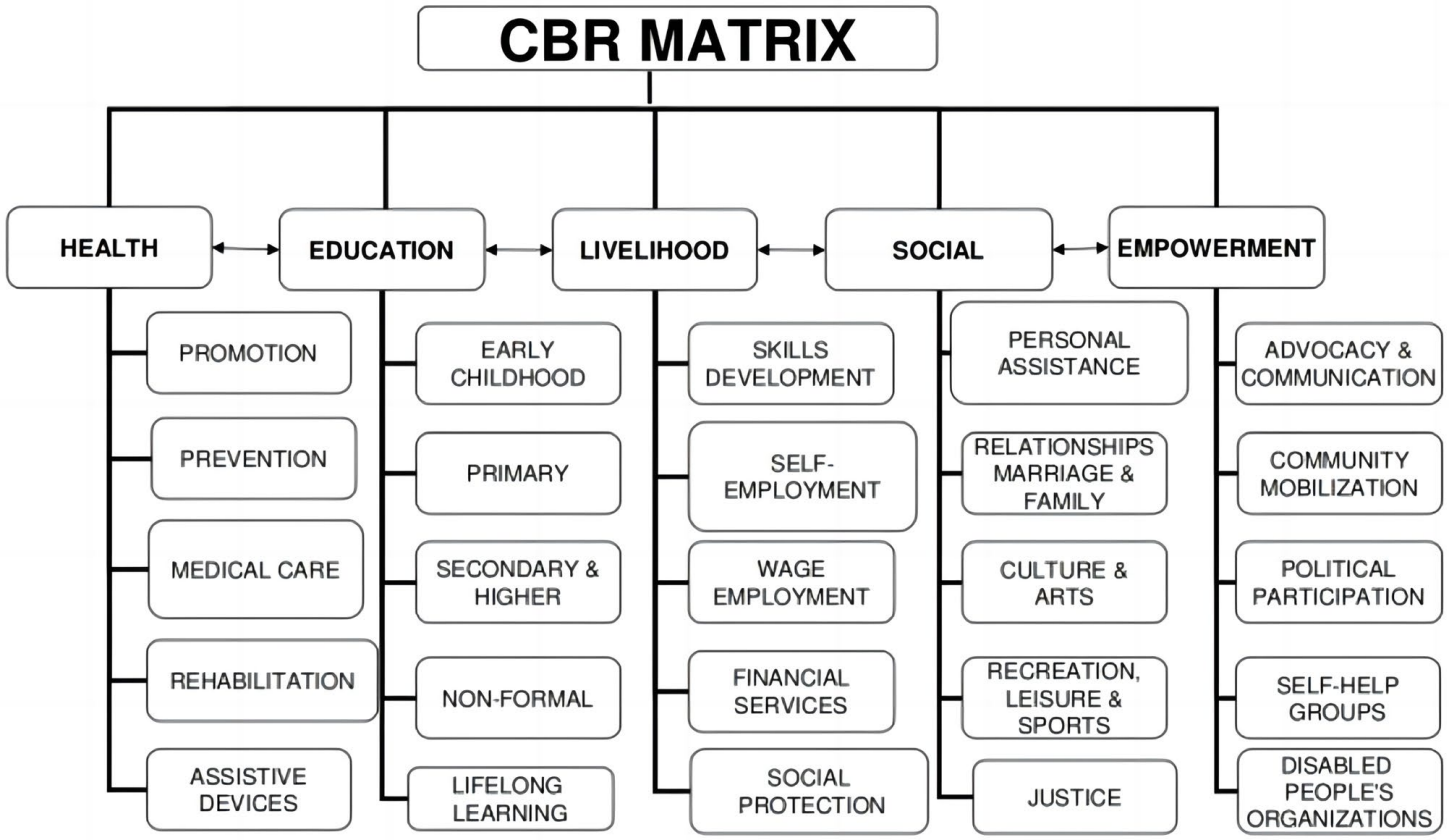
CBR framework

Some frameworks provide descriptions of CBR components. For example, the WHO [10] provides a matrix illustrating the basic framework of CBR that includes five key aspects of individuals: (1) health, (2) education, (3) livelihood, (4) social, and (5) empowerment, as well as subcategories of activities in these key areas (Fig. 1). The International Classification of Functioning, Disability, and Health (ICF) framework states that people’s health functions should be divided into three levels — body function and structure, activity, and participation — all of which interact with personal and environmental factors [11].

The ICF model can be used to evaluate the overall functional status of patients with stroke. Additionally, the American Heart Association (AHA) and American Stroke Association (ASA) guidelines emphasize the importance of rehabilitation programs for patients with stroke, prevention and medical management of comorbidities, body function/structure recovery, activity levels, and patients’ participation in the transition to community rehabilitation [12]. Therefore, we fully consider the influence of the community environment on the rehabilitation process of patients with stroke as well as the role of health, physical function/structure recovery, activity level, and other components in individual functional rehabilitation to better understand the scope and extent of the literature in the field of CBR for patients with stroke.

Although CBR has been implemented in many countries, geographical coverage remains limited [13], and those services are yet to align with the expectations of people with stroke. Due to regional differences in the development of services and the diversity of stroke -related needs, studies report limited access to services, discontinuity of programs and lack of information [14–16]. With the evolution of technology, advanced information systems have been integrated into home-based stroke rehabilitation programs, offering patients convenient access to rehabilitation programs [17, 18]. Nevertheless, the benefits of these emerging technologies still need to be considered on an individualized basis, taking into account factors such as patients’ living environments, practical challenges, and their level of technical proficiency in the home setting [17]. The absence of healthcare workers in the home environment can often lead to a lack of structured therapy, potentially reducing patients’ motivation to participate in rehabilitation programs [17]. In addition, according to the AHA/ASA’s evidence-based recommendations, patients with stroke in the community should receive systematic and multidisciplinary health management services, especially rehabilitation services, to ensure consistency and maximize the effectiveness and efficiency of rehabilitation. This may involve rehabilitation physicians, nurses, physical and occupational therapists, speech-language pathologists, and social workers [12].

Successful stroke rehabilitation is a complex process that requires teamwork among different professional health care providers, and understanding and clarifying their roles and responsibilities is particularly important for effective cooperation among team members and patient participation [16, 19, 20], which can inform the development of a more cost-effective model for CBR services. A previous systematic review explored evidence of the effectiveness of CBR on physical function and activities of daily living (ADL) in patients with stroke [21]; however, to date, no study has yet reviewed the rehabilitation interventions, roles, and functions jointly implemented by multidisciplinary teams in existing CBR programs. Therefore, this study examines the scope of community-based stroke rehabilitation programs involving multidisciplinary teams in existing studies to explore the types of programs and the roles and responsibilities of each program discipline, which is of great significance for the development of systematic and effective stroke CBR service strategies.

## Method

### Design

A scoping review design provides an opportunity to extract the data of various types of research evidence and summarize gaps in the existing evidence. We followed the Joanna Briggs Institute (JBI) methodology [22] and based our review on the normative scoping review framework proposed by Arksey and O’Malley [23] using the Preferred Reporting Items for Systematic Reviews and Meta-Analyses-Extension for Scoping Reviews (PRISMA-ScR) guidelines and checklist [24]. Registration information for this scoping review can be found at osf.io/pv7tg.

### Eligibility criteria

The participants, concepts, and context (PCC) [25] design was considered in the development of the inclusion/exclusion criteria to address the research issues shown in Table 1.

**Table 1:** Study selection criteria (PCC)

	Inclusion	Exclusion
Participants	• Individuals receiving stroke-related CBR programs after discharge from hospital (including those initiated during hospitalization) • Living in a home/community environment • Age ≥ 18	Nil exclusion criteria
Concept	Stroke-related CBR programs: • Implemented by a multidisciplinary team • The role of the implementer is clear • Systematic program model • Cost-effectiveness description	Nil exclusion criteria
Context	• Any country • No date restrictions • Research articles • English or Chinese language • Peer-reviewed	Nil exclusion criteria

### Search strategy

We conducted a preliminary search through PubMed and Medline to analyze the medical subject headings words and free text words related to stroke and CBR intervention research (e.g., stroke, community, rehabilitation) and developed a search strategy. Next, we extended our search to four other English databases (i.e., Web of Science Core Collection (ISI Web of Science), Embase, and Cumulative Index to Nursing and Allied Health Literature (CINAHL) Complete (EBSCO)) and three Chinese databases (i.e., China National Knowledge Infrastructure (CNKI), WangFang, and Vip). The database search was limited to human subjects, and there were no restrictions on date or publication type. Additionally, we only considered studies published in English or Chinese. A completely customized search strategy for PubMed is available in Supplementary Material 1. All retrieval strategies for other databases can be obtained from the first author ZA upon reasonable request.

### Study selection

Two independent reviewers (ZA and KL) conducted an initial review of the titles and abstracts based on the proposed eligibility criteria (Table 1). Any differences in this process were resolved in consultation with a third reviewer (XM). In the case that a consensus was not reached, the study was included in the next phase. Given that the purpose of the scoping review was to explore the breadth, rather than the depth, of the available evidence to address the research questions [22], we did not critically assess the methodological quality of each of the included studies, which would have helped us fully characterize the nature and scope of the evidence.

### Data charting

Retrieval and data extraction were performed between October 2022 and April 2023. The two authors used an extraction table based on the JBI scope definition review template to independently extract and organize the research data, including the country in which the CBR occurred, the target population characteristics, providers and users’ CBR, and the research design. In addition, important information was extracted from the included studies with the agreement of the entire research group, including research features (publication year, location, research design, target population, and research purpose), participant characteristics (age, sex, type of stroke, chronic diseases), CBR provider characteristics and roles, program characteristics (duration, frequency, follow-up time, follow-up mode), outcome characteristics (functional outcomes, body composition, psychosocial outcomes, cardiovascular health), and intervention characteristics (content and delivery model). The intervention characteristics were mapped to the five components of the CBR framework according to their information content, including health promotion, prevention, medical care, and rehabilitation (integrating assistive devices) [10].

## Results

**Fig. 2:**
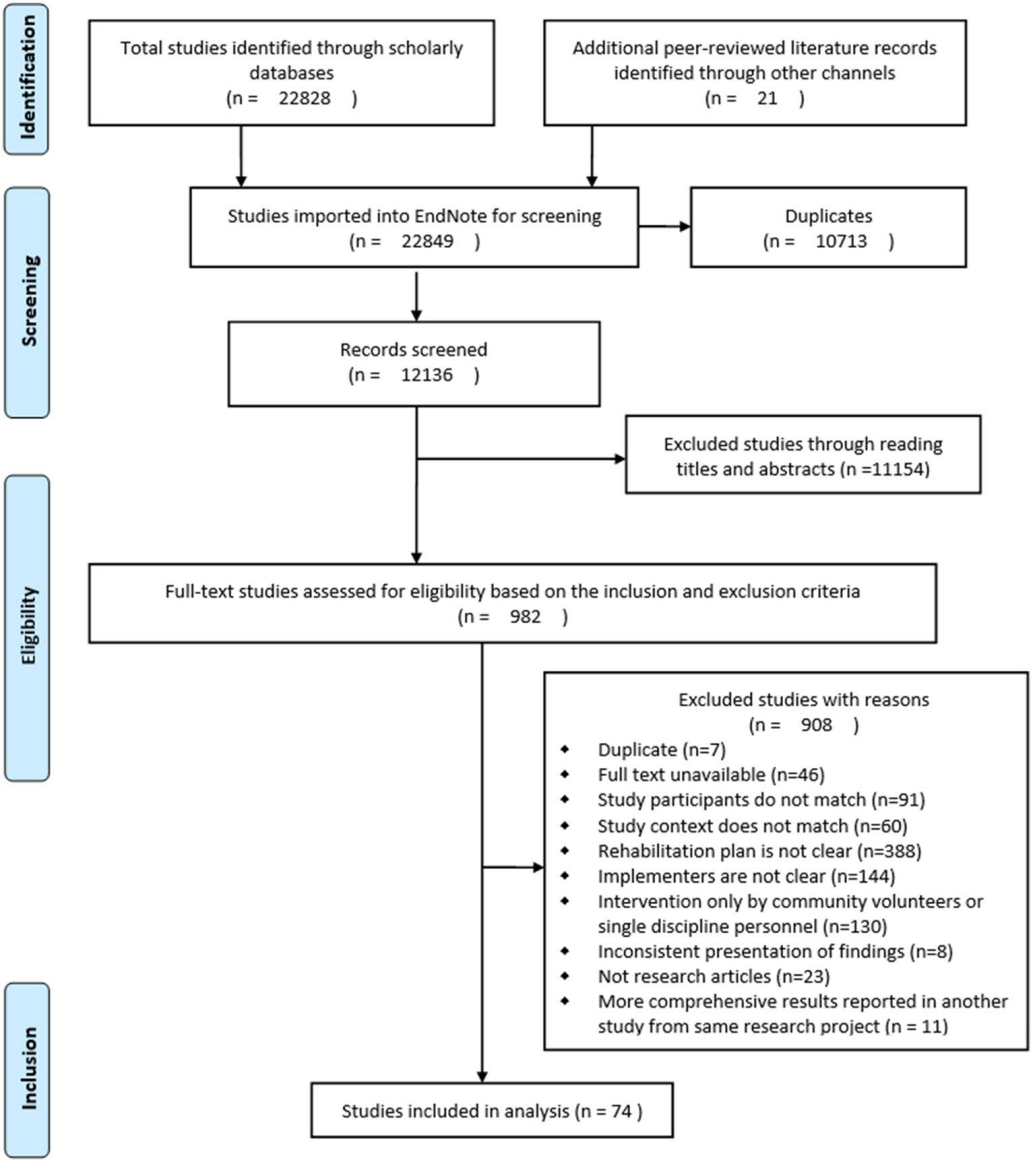
PRISMA flow diagram representing the stages of the scoping review strategy

A total of 22,849 citations were retrieved from the aforementioned databases. After screening for duplicates, 74 studies (67 in Chinese and seven in English) were included in the final review based on the eligibility criteria. Figure 2 shows the details of the search results, duplicate removal, and reasons for exclusion.

### Study characteristics

A total of 74 studies were published between 2008 and 2022. Studies conducted in China accounted for the largest proportion (*n* = 68, 91.89%). The remaining studies were conducted in the United States (*n* = 2, 6.76%) and the United Kingdom, South Korea, France, and Canada (*n* = 1/1/1/1, 1.35%). The majority (52 studies) were randomized controlled trials, 21 were quasi-experimental studies, and one was a retrospective cohort study. For the target population, 64 studies identified the participants as having ischemic and hemorrhagic stroke, whereas others mentioned the type of stroke as ischemic. A total of 12 studies (16.2%) reported the disease stage of the participants, with all participants being in the convalescent period of stroke. A total of 46 studies (62.2%) involved hemiparesis, limb motor dysfunction, dysphagia, and cognitive impairment, whereas the others did not clearly identify functional impairment. Five studies focused on post-stroke depression. A further summary of the study characteristics is provided in Table S1.

### Population characteristics

Table S2 lists the population characteristics of the participants included in the studies, including 6,809 patients with stroke and 49 primary caregivers. The sample size range varied greatly, with a minimum sample size of nine participants and a maximum sample size of 737 participants. The mean age of patients ranged from below 60 years in 17 studies to over 60 years in 48 studies. Five studies only provided the age range without reporting the average age [26–30], and four studies did not report the age characteristics of patients [31–34]. Males accounted for more than three-quarters (76.81%) of the studies. A total of 68.7% (*n* = 41/64) of the studies listed the proportions of ischemic and hemorrhagic stroke, with 35 studies specifying the relevant proportions in the intervention and control groups. Thirteen studies examined coexisting chronic conditions in patients with stroke, including hypertension, diabetes, hyperlipidemia, and coronary heart disease.

### Intervention characteristics

Table S3, S4, and S5 provide the characteristics of each intervention.

### Intervention team

#### Organizational structure

According to the composition of the intervention team, 36 (48.6%) studies involved community and institutional healthcare professionals working together, 28 involved only community healthcare professionals, and ten involved only institutional healthcare professionals. Only 16 studies reported the distribution of team members clearly [26, 35–49]. Seventeen studies (23%) emphasized the joint participation of family members or caregivers in intervention programs [27, 28, 31, 35, 40, 42, 50–60], four studies involved community volunteers/medical social workers [43, 54, 58, 61], and one study reported organizing health education seminars with the assistance of community staff [54].

#### Hospital team

The medical care discipline comprised neurologists (*n* = 26/46) and general practitioners (*n* = 4/46), with one study involving the joint participation of neurologists and general practitioners [45]. Five intervention programs had unclear descriptions of physician categories [38, 41, 53, 62, 63], and 63% (*n* = 29/46) of the intervention programs involved nurse participation. In terms of the composition of the rehabilitation discipline, 13 (27.0%) and 25 (33.8%) intervention programs involved only rehabilitation physicians and rehabilitation therapists, respectively, and only two programs involved both rehabilitation physicians and therapists [41, 61], with only one of these programs describing the type of rehabilitation therapist [61]. Moreover, 12 and eight programs involved psychological discipline [35, 37, 41, 61, 63–70], and nutritionists [35, 38, 55, 65–68, 71], respectively.

#### Community team

The medical care discipline consisted of neurologists (*n* = 2/64) [44, 72], and general practitioners (*n* = 17/64). In 22 intervention programs, the category of physicians was not clearly defined. More than 85.9% (*n* = 55/64) of the intervention programs involved nurse participation. In the rehabilitation discipline, 15 (23.4%) and 12 (18.7%) intervention programs involved rehabilitation physicians and therapists, respectively, with two intervention programs involving joint participation [41, 73]. In one program, the community rehabilitation therapists were clearly identified as physiotherapists, occupational therapists, and speech therapists [74]. Five intervention programs involved the participation of psychological disciplines [28, 51, 59, 60, 75] and public health personnel [28, 50, 60, 76, 77]. None of the programs involved participation of experts in the nutrition discipline.

### Discipline distribution

In all the studies, the disciplines involved in the programs were, in descending order, nursing (*n* = 67, 91.9%), medical care disciplines (*n* = 63, 85.1%), rehabilitation disciplines (*n* = 56, 75.7%), psychology (*n* = 17, 23%), nutrition (*n* = 8, 10.8%), and public health (*n* = 5, 6.7%). Overall, 51 (68.9%) studies involved three or more disciplines: three disciplines (*n* = 39), four disciplines (*n* = 6), and five disciplines (*n* = 6). Of these, the most common combination of multidisciplinary teams was medical care, nursing, and rehabilitation (*n* = 29; 39.2%).

### Roles and responsibilities

#### Hospital team

**Fig. 3:**
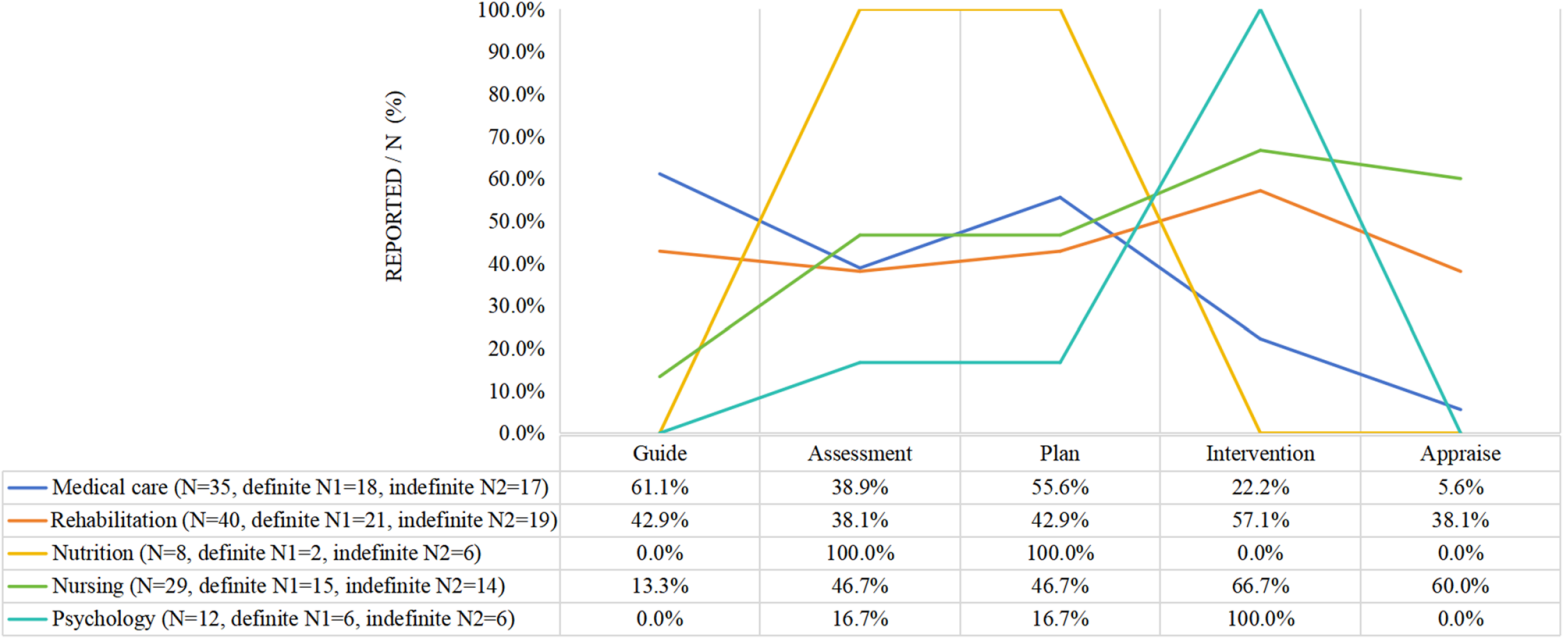
Hospital intervention team member’s role (*N* = 46)

More than 50% of the studies described the responsibilities of the members of their respective disciplines. Physicians were mainly responsible for guiding the entire program and rehabilitation therapists, nurses, nutritionists, and psychologists were mainly responsible for their discipline of implementation (See Fig. 3).

#### Community team

**Fig. 4:**
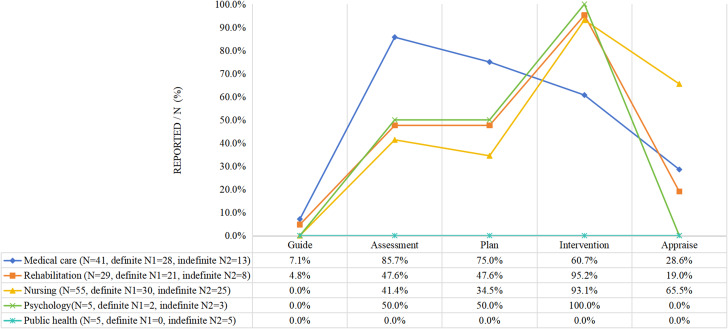
Community intervention team member’s role (*N* = 64)

More than 50% of the studies described the responsibilities of the members of their respective disciplines. The main responsibilities of general practitioners were assessment and evaluation, while rehabilitation therapists, nurses, and psychologists were primarily responsible for implementing the intervention. The duties of the public health discipline team members were not reported (See Fig. 4).

#### Hospital and community team

**Fig. 5:**
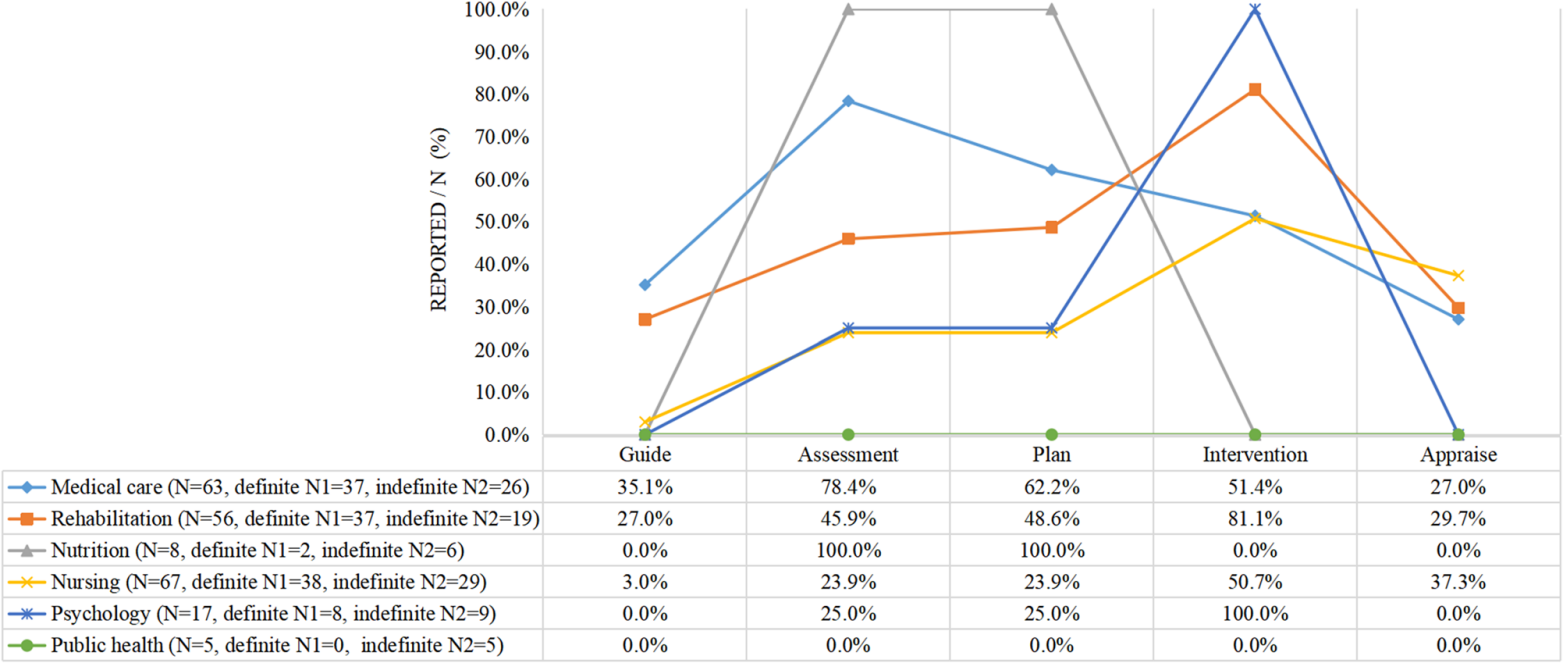
Hospital and community intervention team members’ role (N=74)

Except for the division of responsibilities in public health disciplines that was not reported, the roles of multiple disciplines were indicated in over 50% of the studies, as in the other two groups reported above. Generally speaking, physicians in hospitals and general practitioners in the community are assigned a medical role in which they oversee an entire program, provide guidance, or evaluate outcomes. One study reported that nurses who received rehabilitation training played a role in supervising the intervention process for social workers [54] (See Fig. 5).

**Fig. 6:**
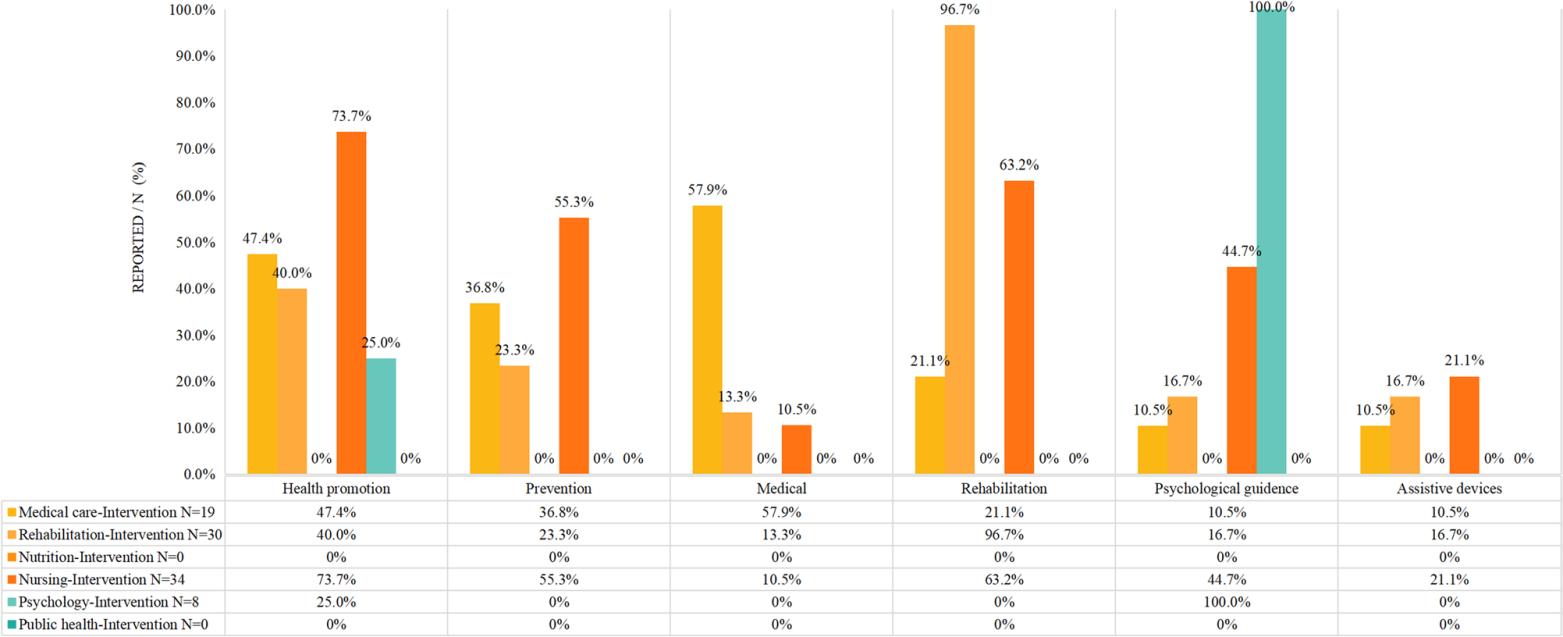
Type of intervention (*N* = 74)

Among the intervention programs that reported responsibilities, the disciplines with the highest contribution of health promotion, prevention, medical care, rehabilitation, psychological guidance, and assistive devices programs were comprised of nurses (73.7%), psychologists (55.3%), physicians (57.9%), rehabilitation therapists (96.7%), psychologists (100%), and nurses (21.1%), respectively (See Fig. 6).

### Intervention time

With the exception of three studies [44, 78, 79] that did not report program duration, the duration of the intervention programs ranged from three weeks to three years, with the vast majority lasting between three and six months (approximately 75.6%); the frequency of the intervention was fixed and did not change over time in approximately 60% of the intervention programs. A total of 48 studies included only pre- and post-intervention assessments and 26 studies reported at least one follow-up assessment, with a maximum of five follow-ups [80]. The follow-up time points ranged from three days to three years after the intervention program.

### Intervention types

Intervention types were categorized into the on-site (*n* = 40, 54.0%) and online intervention modalities (*n* = 26, 35.1%), and eight studies did not clearly describe the location of the intervention [41, 52, 54, 56, 57, 59, 79, 81]. Of the total number of on-site interventions, the primary site was the patient’s home (*n* = 34/66, 51.5%), followed by the “home + community” (*n* = 16/66, 24.2%), “home + community + hospital” (*n* = 5/66, 7.6%), “home + hospital” (*n* = 5/66, 7.6%), community (*n* = 4/66, 6.1%), and “hospital + community” (*n* = 2/66, 3%). Among the online intervention programs, three studies developed a special application to manage the intervention process, and the other 23 studies followed up with subjects using various communication methods, including QQ, WeChat, and phone.

### Intervention content and delivery models

#### Intervention content

Regarding the composition of the intervention content, 71 programs were for rehabilitation (occupational therapy, *n* = 59; daily living skills training, *n* = 55; assistive devices, *n* = 18; speech-language therapy, *n* = 17; swallowing therapy, *n* = 12; cognitive therapy, *n* = 7; physiotherapy, *n* = 7; Chinese medicine, *n* = 7; indefinite, *n* = 9; not applicable, *n* = 3); 53 programs were for health promotion (dietary guidance, *n* = 24; self-monitoring, *n* = 10; psychological guidance, *n* = 43; hygiene, *n* = 9; indefinite, *n* = 2), 40 programs were for medical care (disease knowledge guidance, *n* = 29; psychological treatment, *n* = 11; medication guidance, *n* = 12; outpatient re-examination, *n* = 4), and 38 programs were for prevention (complication prevention, *n* = 27; home environment remodeling, *n* = 25). Of these, only 13 studies involved a single intervention component, including 11 rehabilitation programs [26, 30, 34, 50, 72, 78, 82–86] and two medical programs [37, 59], and more than 82.4% reported 2–4 intervention components in their studies.

Three studies conducted patient rehabilitation training punch card modeling to strengthen patient training compliance [48, 61, 87]. Six studies emphasized that patients should be encouraged to participate in social and recreational activities [35, 54, 62, 64, 83, 88]. Moreover, six studies established a community-centered two-way referral system so that community healthcare professionals could quickly refer patients to hospitals when they had an emergency status [38, 39, 43, 45, 80, 89].

### Delivery models

#### Hospital team

Ten programs were implemented by hospital teams, with home visits, self-study materials, and online Q&A being the main approaches.

#### Community team

A total of 28 programs were implemented by community teams. Home visits, community clinics, and lectures were the main methods used for health promotion, prevention, medical treatment, and rehabilitation. Online Q&As were adopted for medical treatment and rehabilitation.

#### Hospital team and community team

**Fig. 7:**
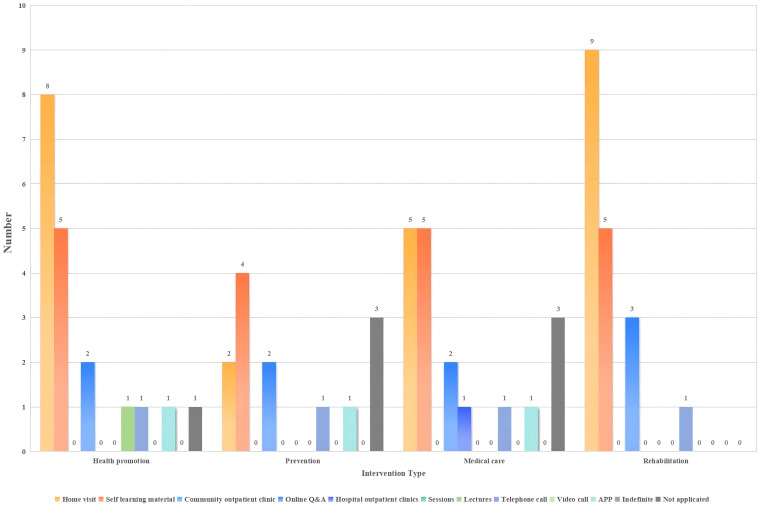
Delivery models for interventions implemented by hospital teams (*N* = 10)

**Fig. 8:**
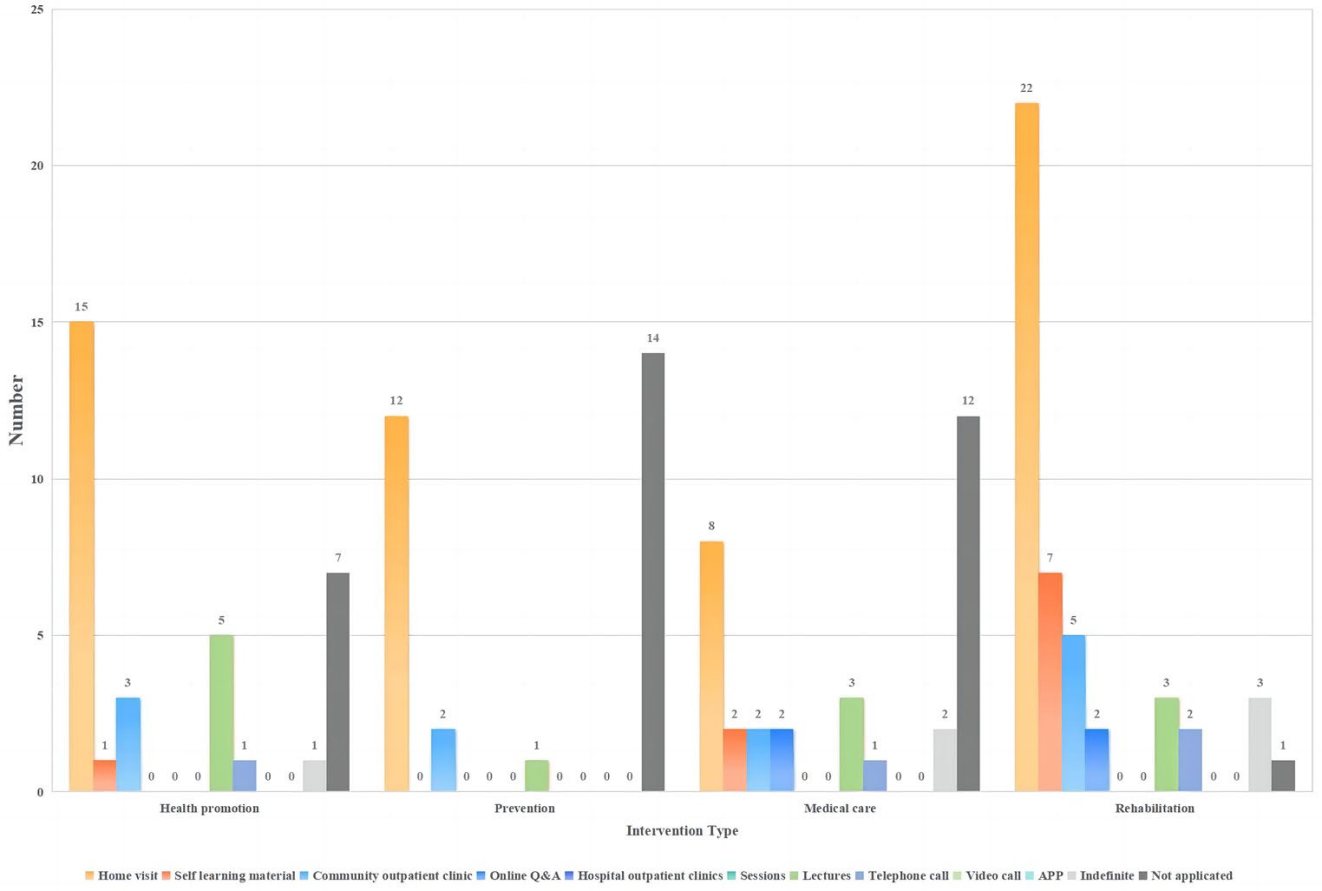
Delivery models for interventions implemented by community teams (*N* = 28)

**Fig. 9:**
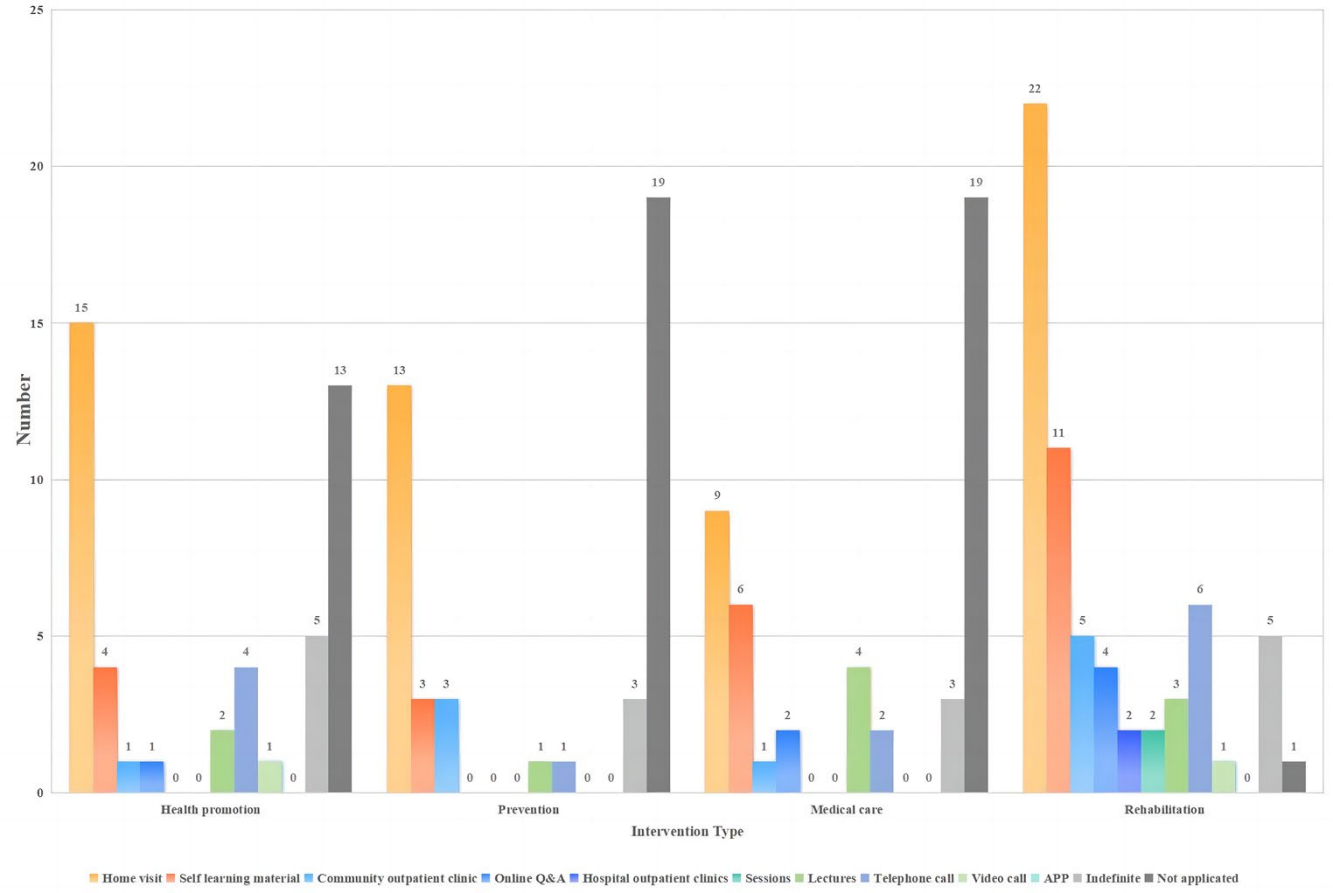
Delivery models for interventions implemented by hospital teams and community teams (*N* = 36)

A total of 36 programs were conducted collaboratively between the hospital and community teams. Home visits, self-learning materials, and telephone calls were the primary delivery models for interventions in the hospital-community team collaboration process. Rehabilitation interventions were the most diverse, whereas prevention interventions were more homogeneous (see Figs. 7, 8 and 9 and Table S6).

### Outcome measures

Table S6 provides a summary of the search results. A total of 42 studies included both physical function and psychosocial measures, with 67 and 49 studies reporting on physical function and psychosocial measures, respectively. Physical function measures included ADLs (59/67 studies), motor functions (24/67 studies), neurological deficits (12/67 studies), balance and fall risk (9/67 studies), walking ability (3/67 studies), muscle strength (2/67 studies), and gait (2/67 studies). The most commonly reported psychosocial outcomes were quality of life (QoL) (23/48 studies), depression (21/48 studies), and anxiety (11/48 studies). Only one study included social participation as an outcome measurement [29]. Four studies reported body composition as the main measurement, including lipid profile and blood glucose levels [36, 47, 60, 90]. Three studies also mentioned cardiovascular outcomes, with cerebral hemodynamics and blood pressure as the primary measures [47, 82, 90].

### Qualitative data

Only one study reported qualitative findings [86] to examine participants’ experiences in the intervention program, and the two themes were (1) confidence through problem-solving, planning, and goal-setting processes and (2) empowerment and emotional support through peer interaction.

## Discussion

We included 74 research articles in this study, with the majority being from China. The studies covered 6,815 patients with stroke and 49 primary caregivers, including both ischemic and hemorrhagic stroke patients. Most study participants resided in urban areas or accessible locations in rural areas, except for those in two studies [80, 91]. Further research may need to consider developing culturally appropriate rehabilitation programs for patients in remote areas, especially for elderly patients living alone, who may have more difficulty accessing rehabilitation services [92].

Three models of CBR programs were found in the included studies: community healthcare professionals only, institutional healthcare professionals only, and a combination of institutional and community healthcare professionals. The combination of institutional and community healthcare professionals was the most common model used for implementing CBR programs. To improve community healthcare services, the Chinese government has developed medical alliances in recent years [93]. With the construction of medical alliances, institutional and community healthcare professionals can work together to provide continued and homogeneous care to patients after discharge. However, the types of healthcare combinations varied among the included studies. Some simply shared patients’ information with the community when they were discharged from the hospital [38, 64], whereas others engaged in comprehensive collaboration among multiple disciplinary groups during the implementation process [45, 55, 65]. Further research is needed to compare the effectiveness of different types of combinations and to provide standardized combination requirements.

Regarding the roles and responsibilities of multidisciplinary team members, usually those in the medical role (e.g., neurologists/rehabilitation physicians/general practitioners) were in charge of the whole program, guiding it, evaluating its implementation, and providing training to community health team members. Other healthcare professionals (e.g., rehabilitation therapists, nurses, nutritionists, and psychologists) were primarily responsible for the implementation of their discipline. In some studies, psychologists were recruited as team members to care for patients’ psychological status after a stroke (Fig. 5). Four studies [43, 54, 58, 61] involved social workers in the intervention, which is important for helping patients return to society effectively [54, 55]. Nursing was the most commonly involved discipline. In the combination of institutional and community healthcare professionals, community health nurses may be the only discipline in the community that provides care for patients after discharge [55, 58, 64, 65, 68, 69, 87]. However, various factors, including daily work overload, lack of stroke-specific knowledge and skills and communication with other healthcare professionals, and undervalued rehabilitation nursing roles may hinder nurses from providing effective rehabilitation nursing services [94, 95]. The number of nurses in China’s primary medical and health institutions has reached 1.15 million, ranking second among all healthcare workers in 2021 [96]. Therefore, we should not only encourage nurses to participate in CBR but also ensure they assume a more dominant role. This can contribute to utilizing medical resources, providing education, and improving self-efficacy and QoL [16, 31, 59].

Regarding the outcome measurements of stroke rehabilitation programs, many studies have focused on multiple functional improvements, the most common being motor function, ADLs, and depression. A few studies have explored the effects of intervention programs on social participation [84–86], which is significant in encouraging patients to maintain social functions and return to their regular social lives. However, as these studies were conducted in the United States or France, the intervention programs may need to be reconsidered by involving different cultural contexts in further research, such as traditional Chinese therapy, Tai Chi, and playing chess [59, 66]. Vocational training was not found in the included studies, which may be due to the fact that the majority of participants were older adults. The major outcome measurements were from the patients or caregivers’ perspectives, including functional improvements, emotional changes, improved QoL, and incidence of complications. The workloads of healthcare professionals were only discussed in one study [84]. Since the CBR programs are long-term intervention programs, a cost-effectiveness analysis for both patients and the healthcare system may need to be conducted in further research, considering such factors as the increased care burden and reduced workforce participation.

### Strengths and limitations

This scoping review is the first to provide an overview of the existing community-based stroke rehabilitation service programs led by multidisciplinary teams. Despite efforts to conduct a comprehensive and systematic literature search, there is a risk of missing relevant studies that should have been identified. First, some studies may have been missed owing to language limitations. Second, because our review was limited to papers published in peer-reviewed journals, we did not examine the grey literature related to this topic, which may have resulted in a limited scoping review. Third, because the scoping review goal was to obtain an overall view rather than to assess the quality of the studies within the field, we did not screen for study quality, which may be one of the reasons for the wide variety of studies in terms of methodology and sample size. Finally, the geographic distribution of the included studies was mainly concentrated in China. Therefore, the CBR programs identified in this review are not immediately transferable to other Western countries as their delivery may vary greatly from country to country due to factors such as socio-economic conditions and cultural issues. Nevertheless, this review has the potential benefit that we can see diversity in the implementation of CBR programs around the world. It is worth noting that our study did not address caregiver/family member experiences in the intervention program as our focus was primarily on patients with stroke. Consequently, the experiences of caregivers/family members were not taken into account in our study design and data collection process. Future research could consider further exploring caregiver/family member roles and experiences in intervention programs to provide a more comprehensive understanding and improve the effectiveness of intervention programs.

## Conclusion

CBR programs show promise in achieving functional and emotional improvements in patients after stroke. The combination of hospital-based and community-based services is a commonly employed model in these programs. Nursing is a key discipline within the CBR program, particularly in the community context, where they play a significant role in implementing the program. However, current evidence remains limited, with inconsistent program availability and outcomes. Further research is required to evaluate the quality of CBR programs and explore the potential for leveraging the role of nurses to enhance stroke services across care settings.

### Electronic supplementary material

Below is the link to the electronic supplementary material.

Supplementary Material 1

Supplementary Material 2

Supplementary Material 3

Supplementary Material 4

Supplementary Material 5

Supplementary Material 6

Supplementary Material 7

## Data Availability

The datasets used and/or analyzed during the current study are available from the first author ZA on reasonable request.

## Abbreviations

CBR: Community-based rehabilitation
JBI: Joanna Briggs Institute
WHO: World Health Organization
ICF: International Classification of Functioning, Disability and Health
AHA: American Heart Association
ASA: American Stroke Association
ADL: Activities of Daily Living
QoL: Quality of Life
PRISMA-ScR: Preferred Reporting Items for Systematic Reviews and Meta Analyses-Extension for Scoping Reviews
PCC: Participants, Concepts, and Context

## References

[1] Collaborators GS (2021). Global, regional, and national burden of stroke and its risk factors, 1990–2019: a systematic analysis for the global burden of Disease Study 2019. Lancet Neurol.

[2] Ma Q, Li R, Wang L, Yin P, Wang Y, Yan C, Ren Y, Qian Z, Vaughn MG, McMillin SE (2021). Temporal trend and attributable risk factors of stroke burden in China, 1990–2019: an analysis for the global burden of Disease Study 2019. Lancet Public Health.

[3] Rimmele DL, Thomalla G (2022). [Long-term consequences of stroke]. Bundesgesundheitsblatt Gesundheitsforschung Gesundheitsschutz.

[4] Feigin VL, Brainin M, Norrving B, Martins S, Sacco RL, Hacke W, Fisher M, Pandian J, Lindsay P (2022). World Stroke Organization (WSO): global stroke fact sheet 2022. Int J Stroke: Official J Int Stroke Soc.

[5] Teasell R, Salbach NM, Foley N, Mountain A, Cameron JI, Jong A, Acerra NE, Bastasi D, Carter SL, Fung J et al. Canadian stroke best practice recommendations: rehabilitation, recovery, and community participation following stroke. Part one: rehabilitation and recovery following stroke; 6th Edition Update 2019. Int J Stroke 2020, 15(7):763–788. 10.1177/1747493019897843 31983296

[6] Mountain A, Patrice Lindsay M, Teasell R, Salbach NM, de Jong A, Foley N, Bhogal S, Bains N, Bowes R, Cheung D (2020). Canadian stroke best practice recommendations: Rehabilitation, Recovery, and Community participation following stroke. Part two: transitions and community participation following stroke. Int J Stroke.

[7] World Health Organization. Report of the International Conference on Primary Health Care, Alma-Ata, USSR, 6-12 September 1978 [report]. 1978. Available from: https://www.who.int/publications/i/item/9241800011 (accessed Jun 2023).

[8] WHO Expert Committee on Disability Prevention and Rehabilitation & World Health Organization. Disability prevention and rehabilitation: report of the WHO Expert Committee on Disability Prevention and Rehabilitation [‎meeting held in Geneva from 17 to 23 February 1981]‎. 1981. Available from: https://iris.who.int/handle/10665/40896 (accessed Jun 2023).

[9] International Labour Organization, United Nations Educational, Scientific and Cultural Organization, World Health Organization. CBR: a strategy for rehabilitation, equalization of opportunities, poverty reduction and social inclusion of people with disabilities: joint position paper. World Health Organization. 2004. Available from: https://iris.who.int/handle/10665/43060 (accessed Jun 2023).

[10] Khasnabis C, Heinicke Motsch K, Achu K, Al Jubah K, Brodtkorb S, Chervin P, Coleridge P, Davies M, Deepak S, Eklindh K, et al. Community-Based Rehabilitation: CBR Guidelines. Geneva: World Health Organization; 2010. 26290927

[11] World Health Organization. International classification of functioning, disability and health: ICF. World Health Organization. 2001 Available from: https://iris.who.int/handle/10665/42407 (accessed Jun 2023).

[12] Winstein CJ, Stein J, Arena R, Bates B, Cherney LR, Cramer SC, Deruyter F, Eng JJ, Fisher B, Harvey RL (2016). Guidelines for adult stroke rehabilitation and recovery: a guideline for healthcare professionals from the American Heart Association/American Stroke Association. Stroke.

[13] Iemmi V, Blanchet K, Gibson LJ, Kumar KS, Rath S, Hartley S, Murthy GVS, Patel V, Weber J, Kuper H (2016). Community-based rehabilitation for people with physical and mental disabilities in low- and middle-income countries: a systematic review and meta-analysis. J Dev Eff.

[14] Guo YF, Zhang ZX, Lin BL, Mei YX, Liu QX, Zhang LY, Wang WN, Li Y, Fu ZR. The unmet needs of community-dwelling stroke survivors: a systematic review of qualitative studies. Int J Environ Res Public Health 2021;18(4): 2140. 10.3390/ijerph18042140 PMC7926407 33671734

[15] Pindus DM, Mullis R, Lim L, Wellwood I, Rundell AV, Abd Aziz NA, Mant J (2018). Stroke survivors’ and informal caregivers’ experiences of primary care and community healthcare services- a systematic review and meta-ethnography. PLoS ONE.

[16] Dalvandi A, Ekman SL, Khankeh HR, Maddah SS, Heikkilä K (2012). Rehabilitation experts’ experience of community rehabilitation services for stroke survivors in Iran. Top Stroke Rehabil.

[17] Chen Y, Abel KT, Janecek JT, Chen Y, Zheng K, Cramer SC (2019). Home-based technologies for stroke rehabilitation: a systematic review. Int J Med Informatics.

[18] Sarfo FS, Ulasavets U, Opare-Sem OK, Ovbiagele B (2018). Tele-rehabilitation after stroke: an updated systematic review of the literature. J Stroke Cerebrovasc Diseases: Official J Natl Stroke Association.

[19] Magwood GS, Nichols M, Jenkins C, Logan A, Qanungo S, Zigbuo-Wenzler E, Ellis C (2020). Community-based interventions for Stroke provided by nurses and Community Health Workers: a review of the literature. J Neurosci Nurs.

[20] Davoody N, Koch S, Krakau I, Hägglund M (2016). Post-discharge stroke patients’ information needs as input to proposing patient-centred eHealth services. BMC Med Inf Decis Mak.

[21] Zeng X, Balikuddembe JK, Liang P. Impact of community-based rehabilitation on the physical functioning and activity of daily living of stroke patients: a systematic review and meta-analysis. Disabil Rehabil 2023;45(3):403-414. 10.1080/09638288.2022.2037755 35200068

[22] Peters M, Godfrey C, Khalil H, McInerney P, Parker D, Soares C. Guidance for conducting systematic scoping reviews. Int J Evid Based Healthc 2015; 13(3):141-146. 10.1097/XEB.0000000000000050 26134548

[23] Arksey H, O’Malley L (2005). Scoping studies: towards a methodological framework. Int J Soc Res Methodol.

[24] PRISMA Extension for Scoping Reviews (PRISMA-ScR) (2018). Checklist and Explanation. Ann Intern Med.

[25] Stern C, Jordan Z, McArthur A (2014). Developing the review question and inclusion criteria. Am J Nurs.

[26] Chen S. Effect of the hospital-community linkage nursing management mode on the rehabilitation and quality of life in patients with stroke. J Bengbu Med Coll 2018;43(1):110-113.

[27] Huang CX, Zhao SH. The impact of process management on quality control of community care for stroke patients. Chin Gen Pract 2011;14(35):4028-4031.

[28] Xue B, Tang ZY, Liu TL, Zhao S, Qin D, Gu WQ (2016). Effects of home rehabilitation in a community team model on physical function and activities of daily living in stroke patients. Shanxi Med J.

[29] Yu J, Hu Y, Wu Y, Chen W, Zhu Y, Cui X, Lu W, Qi Q, Qu P, Shen X (2009). The effects of community-based rehabilitation on stroke patients in China: a single-blind, randomized controlled multicentre trial. Clin Rehabil.

[30] Park Y-J, Lee C-Y (2016). Effects of community-based rehabilitation program on activities of daily living and cognition in elderly chronic stroke survivors. J Phys Therapy Sci.

[31] Dan SC, Gao L, Ge XH, He Q. Effect of Community Rehabilitation on activity of daily living in stroke patients. Heilongjiang Med J 2015;28(4):887-889.

[32] Li GZ. Effectiveness of Orem’s self-care theory in the community care of stroke patients. Chin J Trauma Disabil Med 2014;22(6):273-274.

[33] Zhu JX, Zhou FY, Deng HD, Li YL. The role of home beds in community-based rehabilitation of patients recovering from stroke. Chin J Clin 2014;42(6):41-42.

[34] Xue B, Gu WQ, Tang ZY, Liu TL, Zhao S, Qin D. Study of the impact of the family rehabilitation on the life quality, anxiety and depression of the patients with the stroke under the community team mode. Shanghai Med Pharm J 2015;36(20):60-62.

[35] Wang JJ, Xie P, Bai JX, Cai GL. Impact of hospital-community-home continuity of care on stroke patients with non-dementia cognitive impairment. Today Nurse 2022;29(2):84-87.

[36] Zhang WF, Chen LN. Effects of mobile phone app based extended nursing care on the stroke patients in community. Chin J Mod Nurs2018;24(2):190-195.

[37] Chen RX, Guan ZJ, Lu YX, Huang KF, Deng LM, Chen SY. Impact of a combined hospital-community-home care model on the quality of life of depressed patients after stroke. Int Med Health Guidance News 2012;18(15):2166-2168.

[38] Fu XM, Jin SJ, Zeng XL, Chen Y. Effect of home pension system under medical-nursing combined model on quality of life in elderly patients with stroke. Hainan Med J 2019;30(10):1352-1355.

[39] Chen RX, Guan ZJ, Wu YE, Fang YG, Wei YL, Fang YH. Effects of care provided by hospital, community and family on quality of life of stroke patients. J Nurs Sci 2012;27(8):82-84.

[40] He Y. Exploring the effectiveness of community-based rehabilitation guidance in stroke. Sci Tech Inform Gansu 2015;44(6):116-117.

[41] Gao SF, Sun PY, Jiao LQ. Effects of different community rehabilitation models on daily living ability and neuropsychology of patients with cerebral infarction. Chinese Journal of Integrative Medicine on Cardio-/Cerebrovascular Disease 2013;11(7):826-827.

[42] Hu XX, Li H. Effect of community care and family members participation on rehabilitation of patients with cerebral infarction. Mod Clin Nurs 2016;15(5):26-30.

[43] Zhang Y, Wang L, Liu Y, Gao Y, Qian F, Wang YQ. Application effects of community elderly health service model in home health management of stroke patients. Chin Nurs Manage 2022;22(3):334-338.

[44] Wu L. Effective application of community rehabilitation nursing in the rehabilitation management of patients with sequelae of cerebral infarction. Yi Shou Bao Dian 2020;(10):0058.

[45] Yu XY, Sang SH, Chi J, Du X, Ren QH, Zhang L. Application of information-based medical collaborative management and family cooperative management in stroke patients taking rehabilitation at home. Int Med Health Guidance News 2022;28(11):1524-1529.

[46] Meng YQ, Liu GJ, Fan ZC, Qi H, Bao S RL, Zhu RX. Application of remote scientific rehabilitation guidance and education in hemiplegia rehabilitation. Int Med Health Guidance News 2022;28(15):2127-2131.

[47] Wang L (2005). Community specialist intervention and functional prognosis in patients recovering from stroke. Chin J Clin Rehabilitation.

[48] Chen WP, Lin D, Lu W. A study on the application of functional gait training in a community-based realistic environment in elderly patients in the rehabilitation period after stroke. J Nurs Rehabilitation 2020;19(5): 65-68.

[49] Meng FY, Wang Y. Influence of continuity nursing on self-care ability of cerebral apoplexy patients in community during rehabilitation. Chin Nurs Researsh 2015; 29(6): 2215-2218.

[50] Lan Q, Ji ML, Chen LQ. A study of the effectiveness of stroke home rehabilitation team interventions for patients in the community. Shanghai Nurs 2008;8(4): 34-36.

[51] Zhang XQ, Wang C, Bi ZZ, Gu YM, Liu YL, Jin LJ, Jin RX, Chen LB. Effect of the community-based rehabilitation unit on stroke: a prospective randomized control study. Shanghai Med Pharm J 2015;36(12): 47-50.

[52] Huang WL, He YJ. Influence of quantitative nursing intervention on rehabllitation of community stroke patients. Mod Hosp 2012;12(7): 151-153.

[53] Rao R, Ye D, Hu J. A study of out-of-hospital acceptance of continuity of care services for patients recovering from stroke. Chin J Rehabilitation 2014;29(6): 453-454.

[54] Liu Y, Liu JJ, Jin JP. The efficacy of the three-society linkage model for community-based rehabilitation of stroke patients. Chin J Gerontol 2019;39(5): 1051-1053.

[55] Li H. Hospital-community-family interface continuity of care in patients recovering from stroke. Shanxi Med J 2020;49(13): 1748-1750.

[56] Li L, Li SW, Zhao H, Pan H, Zhang L, Han S, Zhao L, Wu GS, Mao JF, Li Y. Effect evaluation of the application of community rehabilitation pathway in home rehabilitation. Heilongjiang Med J 2014;38(11): 1331-1334.

[57] Cao QY, Zhong HM, Liu QE, Zhang HM, Liao YY. Application of group therapy in social rehabilitation training on stroke patients. China Med Pharm 2013;3(11): 47-48.

[58] Liu SS. Take the family as the influence of self-management mode on rehabilitation of patients with cerebral apoplexy. China Health Ind 2015;12(8): 142-144.

[59] Liang N, Wang ZK, Zhang ZW, Chen D, Lu XJ, Song X, Chen RQ, Hu YY, He QC, Qin QQ. Influence of community psychological intervention based on five-element theory on neural function in patients with post-stroke depression. Intern Med 2016;11(2): 174-176.

[60] Liu CF, Zhang J, Li XX, Chen YR, Zhang ZX, Sun XY, Miao YZ, Wang LL. Effect of collaborative rehabilitation intervention on functional recovery and quality of life in elderly stroke patients. Chin J Practical Nerv Dis 2022;25(2): 197-201.

[61] Shi SX, Xu WW, Liu X, Huang J, Guo Y, Fu L, Jin H. Study on the efficacy of community-led home rehabilitation model on stroke patients under the background of internet plus. J Mod Med Health 2022;38(17): 2904-2907.

[62] Zhang XC, Li SH, Qian YF, Wang LQ, Yu B. Observation on the effect of home care knowledge training for elderly people recovering from stroke in the community. J Nurs(China) 2010;17(11): 65-67.

[63] Jiao LQ, Gao SF, Sun PY. A clinical study of comprehensive community-based rehabilitation for stroke hemiparesis directed by a general hospital. Chin J Integr Med Cardio-/Cerebrovascular Disease 2013;11(10): 1218-1219.

[64] Chen L. The effect of hospital-community-family rehabilitation nursing model on the psychological state and daily living ability of patients with cerebral infarction. Heilongjiang J Traditional Chin Med 2021;50(3): 232-233.

[65] Cai LN, Shi JL, Chen XL. Effectiveness of hospital-community-family extended care in elderly patients with ischemic stroke. Nurs Integr Traditional Chin Western Med 2021;7(8): 136-138.

[66] He ML, Xie YH, Wang WH, Li HS. The effect of community-based home rehabilitation care model on improving the psychological status of stroke patients. J Qilu Nurs 2019;25(3): 105-107.

[67] Li XP, Wang L, Lan YL, Huang WD, Zhang Q. Impact of community home-based rehabilitation nursing on psychological state of cerebral apoplexy patients in urban communities. Chin Nurs Researsh 2010;24(3): 838-839.

[68] Li L, Yue P, Zhang Y. The effect of hospital-community-family rehabilitation nursing model on medical compliance behavior and daily living ability of hemiplegic patients with cerebral infarction. Henan Med Res 2019;28(5): 913-915.

[69] Chen Q, Shi HQ, Wu ZH, Zhi BH, Shi MF. The role of hospital-community-family extended rehabilitation nursing intervention in improving physical function and psychological status of stroke patients. Chronic Pathematology J 2018; 19(2): 228-230.

[70] Qin Y, Li XP, Wang L. Observation on the effect of community rehabilitation nursing care for patients with post-stroke sequelae. J Nurs(China) 2011;18(6): 63-65.

[71] Li XP, Wang L, Zhang Q, Huang WD, Lai GF. Effectiveness study of nursing by rehabilitation collaboration network among stroke family in urban community. J Nurses Train 2011;26(9): 773-776.

[72] Hu SH, Ling Q, Xu J, Jiang LJ, Lu Y, Su N, Hu JQ, Zhang XF, Shen MH, Li RY. Intervention effect of community rehabilitation model in stroke patients based on regional medical association. Chin Gen Pract 2016;19(22): 2729-2733.

[73] Mao JB, Hu HJ, Zhang JM. Influence of community family doctor as the center on rehabilitation of functional recovery of patients with stroke. Shanghai Med Pharm J 2018;39(8): 60-62.

[74] Liu HL, Zhou B, Zhao Z, Yang Y, Lv XQ, Wang Y, Yu T. Home-based telerehabilitation guidance for stroke patients. Chin J Rehabilitation Theory Pract 2021;27(7): 807-811.

[75] Chen W, Jiang B, Zhu HX, Yang ZJ, Xu GZ, Peng L, Wei J. The effect of rehabilitation of family doctor team intervention on community post-stroke depression patients. Shanghai Med Pharm J 2019; 40(10): 56-58.

[76] Wei XP, Yu LM, Hu W. Observation of curative effects on treatment of patients with limb disability sequela after stroke in community health service. Journal of Neurology and Neurorehabilitation 2009;6(3): 191-193.

[77] Zhang XQ, Wang C, Bi ZZ, Gu YM, Jin LJ, Jin RX, Chen LB, Liu YL. Establishment of the community stroke rehabilitation unit and evaluation of its operational result. Shanghai Med Pharm J 2015;36(4): 57-59.

[78] Wu LB. An analysis of the effects of community-based rehabilitation for stroke patients. Contemp Med Symposium 2017;15(10): 59-60.

[79] Li XM. Effectiveness of community-based rehabilitation therapy in improving activities of daily living of stroke patients. Biped and Health 2017;26(24): 59-60.

[80] Wu MH, Zhu CP, Xu XF, Lu AM, Chu HF. Effect of home-based rehabilitation nursing on the ability of activities of daily living in the rural patients with stroke. Shanghai Med Pharm J 2017;38(22): 60-62.

[81] Jiang MH, Qin B, Chen QG. The effect of community rehabilitation on the quality of life of homebound stroke patients with hemiplegia. Nurs Pract Res 2010;7(13): 117-119.

[82] Liao QH, Wang F, Xu WW, Zhi JF, Chen SL, Li J (2019). Impact of a community-based stroke rehabilitation model on neurological rehabilitation in recovery from cerebral infarction. Zhejiang Clin Med J.

[83] Gao CH, Huang XL, Zhang W, Cai JH, Liu YL, Wang W. The effects of core stability training on stroke patients’ motor function. Stroke Nerv Dis 2014;21(4): 207-211.

[84] Daviet JC, Compagnat M, Bonne G, Maud L, Bernikier D, Salle JY. Individualized home-based rehabilitation after stroke in France: a pragmatic study of a community stroke rehabilitation team. Can J Neurol Sci 2023;50(3): 405-410. 10.1017/cjn.2022.26 35477586

[85] Eng JJ, Chu KS, Kim CM, Dawson AS, Carswell A, Hepburn KE (2003). A community-based group exercise program for persons with chronic stroke. Med Sci Sports Exerc.

[86] Lee D, Fischer H, Zera S, Robertson R, Hammel J (2017). Examining a participation-focused stroke self-management intervention in a day rehabilitation setting: a quasi-experimental pilot study. Top Stroke Rehabil.

[87] Yang Y, Ma YH, Zhao Z, Zhou B, Liu HL (2019). Study on the advantages and effects of applying community-based tele-rehabilitation for patients with cerebral infarction. World Latest Med Inform.

[88] Lv QF. Analysis of the effect of home care knowledge training in community-based elderly people recovering from stroke. Reflexology Rehabilitation Med 2020;(9): 167-168.

[89] Zhang XM, Zhu WH, Jiang QK, Gan LF. Efficiency of community and family-based rehabilitation strategy in the rehabilitation of post-stroke depression patients. Chin J Clin Med 2020;27(4): 657-661.

[90] Moore SA, Hallsworth K, Jakovljevic DG, Blamire AM, He JB, Ford GA, Rochester L, Trenell MI (2015). Effects of community exercise therapy on metabolic, brain, physical, and cognitive function following stroke: a randomized controlled pilot trial. Neurorehabilit Neural Repair.

[91] Cao QR, Feng SW, Huang SY, Li XY, Lin XY, Tan YM, Xu CL. Study of home rehabilitation model and effectiveness analysis on stroke patients in rural area. Chin J Rehabilitation Theory Pract 2016;31(3): 190-192.

[92] Qian J, Liu Z, Li Y, Qian H, Li M, Zhang Z (2023). Longitudinal study on the trajectory of frailty and its influencing factors in rural senile stork patients. Chin J Practical Nerv Dis.

[93] Nie Z. Research on the construction status and optimization countermeasures of primary medical and health institutions in the medical alliance-Taking Z community health service center in Shandong Province as an example. Master’s Thesis Shandong University; 2022.

[94] Meng X, Chen X, Liu Z, Zhou L (2020). Nursing practice in stroke rehabilitation: perspectives from multi-disciplinary healthcare professionals. Nurs Health Sci.

[95] Tanlaka EF, McIntyre A, Connelly D, Guitar N, Nguyen A, Snobelen N (2023). The role and contributions of nurses in Stroke Rehabilitation Units: an integrative review. West J Nurs Res.

[96] National Bureau of Statistics of China. China Health Statistics Yearbook 2022. National Bureau of Statistics of China. 2022. Available from: https://www.stats.gov.cn/sj/ndsj/2022/indexeh.html (accessed Jun 2023).

