# Supplementary material for: Community-based rehabilitation services implemented by multidisciplinary teams among adults with stroke: a scoping review with a focus on Chinese experience
Source: BMC Public Health. 2024 Mar 7;24:740. doi: 10.1186/s12889-024-18218-1 (PMC10921794; doi:10.1186/s12889-024-18218-1)
Supplement: Supplementary file 1 — Supplementary Material 1 [file 12889_2024_18218_MOESM1_ESM.pdf]

Search strategy:

#1 "Stroke"[MeSH Terms] OR "Stroke"[Title/Abstract] OR "Cerebrovascular Accident"[Title/Abstract]
#2 "Community Health Planning"[MeSH Terms] OR "Community Integration"[MeSH Terms] OR "Therapeutic Community"[MeSH Terms] OR "Community Participation"[MeSH Terms] OR "Community-Based Participatory Research"[MeSH Terms] OR "Community Support"[MeSH Terms] OR "Community Health Planning"[Title/Abstract] OR "Community Integration"[Title/Abstract] OR "Therapeutic Community"[Title/Abstract] OR "Community Participation"[Title/Abstract] OR "Community-Based Participatory Research"[Title/Abstract] OR "Community Support"[Title/Abstract] OR "community"[Title/Abstract] OR "community-based"[Title/Abstract] OR "community-delivered"[Title/Abstract] OR "community engagement"[Title/Abstract] OR "community program"[Title/Abstract] OR "community role"[Title/Abstract]
#3 "rehabilitation, vocational"[MeSH Terms] OR "Rehabilitation"[MeSH Terms] OR "Stroke Rehabilitation"[MeSH Terms] OR "Telerehabilitation"[MeSH Terms] OR "rehabilitation vocational"[Title/Abstract] OR "Rehabilitation"[Title/Abstract] OR "Habilitation"[Title/Abstract] OR "Stroke Rehabilitation"[Title/Abstract] OR "Service"[Title/Abstract] OR "model"[Title/Abstract] OR "Intervention"[Title/Abstract] OR "Telerehabilitation"[Title/Abstract] OR "tele-assisted"[Title/Abstract] OR "tele-health"[Title/Abstract]
#4 ("Recovery of Function"[Mesh] OR "Motor Activity"[Mesh] OR "Patient Participation"[Mesh] OR "Comparative Effectiveness Research"[Mesh] OR "Cost-Benefit Analysis"[Mesh] OR "Cost-Benefit Analyses"[Mesh] OR "Treatment Outcome"[Mesh]) OR ("Outcome"[Title/Abstract] OR "Function*"[Title/Abstract] OR "effectiveness"[Title/Abstract] OR "Activit*"[Title/Abstract] OR "Participation"[Title/Abstract] OR "Cost Effectiveness Analysis"[Title/Abstract] OR "feasib*"[Title/Abstract] OR "efficacy"[Title/Abstract])

#5 #1 AND #2 AND #3 AND #4
