# Supplementary material for: Community-based rehabilitation services implemented by multidisciplinary teams among adults with stroke: a scoping review with a focus on Chinese experience
Source: BMC Public Health. 2024 Mar 7;24:740. doi: 10.1186/s12889-024-18218-1 (PMC10921794; doi:10.1186/s12889-024-18218-1)
Supplement: Supplementary file 2 — Supplementary Material 2 [file 12889_2024_18218_MOESM2_ESM.docx]

Table S1 Characteristics of included studies (N=74)

| **Author(s)** | **Publication year** | **Country** | **Location** | **Study design** | **Randomization** | **Target population** | **Study aim** |
| --- | --- | --- | --- | --- | --- | --- | --- |
| (Wang et al., 2022) | 2022 | China | Yangzhou, Jiangsu Province | Quasi-experimental study (admission order grouping) | NA | Stroke patients with cognitive impairment without dementia | To explore the effect of hospital-community-family continuing nursing on clinical rehabilitation of patients with non-dementia cognitive impairment after stroke. |
| (Zhang and Chen, 2018) | 2018 | China | Wuxi, Jiangsu Province | Prospective RCT | Random number table | Stroke patients | To explore the effects of transitional care based on mobile App on motor function, self-efficacy, quality of life and physiological indexes of stroke patients in community. |
| (Lan et al., 2008) | 2008 | China | Shanghai | Quasi-experimental study (grouping by different Communities) | NA | Stroke patients (limb dysfunction) | To explore the effects of community rehabilitation team on the rehabilitation of stroke patients. |
| (Chen, 2021) | 2021 | China | Xinxiang, Henan Province | Prospective RCT | Random number table | Ischemic stroke patients | To explore the effects of hospital-community-family rehabilitation nursing intervention in patients with cerebral infarction. |
| (Zhang et al., 2015) | 2015 | China | Shanghai | Prospective RCT | Random number table | Stroke patients (limb dysfunction) | To explore the effects of community stroke rehabilitation unit model on stroke patients. |
| (Jiang et al., 2010) | 2010 | China | Nanning, Guangxi Zhuang Autonomous Region | Prospective RCT | NR | Stroke patients with hemiplegia | To explore the effects of rehabilitation on the quality of life of stroke patients with hemiplegia at home |
| (Chen et al., 2012) | 2012 | China | Dongguan, Guangdong Province | Quasi-experimental study (grouping by odd or even number of hospitalization ID) | NA | Patients with post-stroke depression | To explore the effects of hospital, community and home combined care model on the quality of life of patients with post-stroke depression. |
| (Chen, 2018) | 2018 | China | Jinzhou, Liaoning Province | Prospective RCT | NR | Stroke patients | To explore the effects of hospital-community linked nursing management model on functional improvement and quality of life of stroke patients. |
| (Cai et al., 2021) | 2021 | China | Xiamen, Fujian Province | Prospective RCT | Random number table | Patients with ischemic stroke | To explore the effects of "hospital-community-family" linkage continuous nursing in elderly patients with ischemic stroke. |
| (Fu et al., 2019) | 2019 | China | Haikou, Hainan Province | Prospective RCT | Random number table | Senile stroke patients | To explore the effects of "combination of medical care and nursing" home care service system on the quality of life of elderly patients with stroke. |
| (Chi, 2009) | 2009 | China | Taizhou Zhejiang Province | Quasi-experimental study (before and after control) | NA | Stroke patients with hemiplegia | To investigate the effects of motor function rehabilitation training on the improvement of motor function in 9 stroke patients in community |
| (Wu et al., 2017) | 2017 | China | Shanghai | Prospective RCT | Random number table | Rural stroke patients (limb dysfunction) | To explore the effects of family rehabilitation nursing intervention based on general practice team model on activities of daily living in rural patients after stroke. |
| (Chen et al., 2012) | 2012 | China | Dongguan, Guangdong Province | Quasi-experimental study (grouping by odd or even number of hospitalization ID) | NA | Stroke patients | To explore the effects of hospital-community-home combined care model on rehabilitation and quality of life of stroke patients. |
| (Zhang et al., 2020) | 2020 | China | Shanghai | Prospective RCT | Random number table | Patients with post-stroke depression | To evaluate the effects of community-family rehabilitation model on the rehabilitation of patients with post-stroke depression (PSD). |
| (Dan et al., 2015) | 2015 | China | Xuzhou, Jiangsu Province | Prospective RCT | NR | Stroke patients (limb or dysphagia dysfunction) | To explore the effects of community and family rehabilitation intervention on functional recovery and daily living ability of stroke patients. |
| (He, 2015) | 2015 | China | Lanzhou, Gansu Province | Quasi-experimental study (grouping by patients’ wishes) | NA | Stroke patients (limb dysfunction) | To investigate the effects of community medical guidance model on the rehabilitation of stroke patients. |
| (Huang and He, 2012) | 2012 | China | Guangzhou, Guangdong Province | Prospective RCT | NR | Patients with post-stroke depression and limb dysfunction | To explore the effects of quantitative nursing intervention on rehabilitation treatment of stroke patients in community. |
| (Rao et al., 2014) | 2014 | China | Wuhan, Hubei Province | Quasi-experimental study (before and after control) | NA | Stroke patients (convalescent period) | To explore the effects of continuous nursing service outside the hospital on patients with stroke. |
| (Wei et al., 2009) | 2009 | China | Shanghai | Quasi-experimental study (admission order grouping) | NA | Stroke patients (limb dysfunction) | To evaluate the long-term effects of community-based rehabilitation on the sequelae of physical disability among stroke patients. |
| (Gao et al., 2013) | 2013 | China | Beijing | Prospective RCT | Random number table | Stroke patients (limb dysfunction) | To explore the effects of different community rehabilitation treatment models on activities of daily living and neuropsychology in patients with cerebral infarction. |
| (Shi et al., 2022) | 2022 | China | Hefei, Anhui Province, | Quasi-experimental study (admission order grouping) | NA | Stroke patients | To explore the effects of community-led home rehabilitation mode on motor function, activity of daily living, quality of life and anxiety in stroke patients. |
| (Liu et al., 2019) | 2019 | China | Shanghai | Prospective RCT | Random number table | Stroke patients | To explore the efficacy of the "community government, community health service center, social welfare organization (social worker)" model for community rehabilitation of stroke patients. |
| (Li et al., 2011) | 2011 | China | Nanning, Guangxi Zhuang Autonomous Region | Prospective RCT | NR | Stroke patients | To analyze the effectiveness of rehabilitation nursing intervention of community and family rehabilitation cooperation network for stroke patients in urban China. |
| (Hu and Li, 2016) | 2016 | China | Foshan, Guangdong Province | Quasi-experimental study (before and after control) | NA | Ischemic stroke patients (limb dysfunction) | To explore the influence of community rehabilitation nursing on the rehabilitation of cerebral infarction patients. |
| (Li, 2014) | 2014 | China | Nanyang, Henan Province | Quasi-experimental study (grouping by the order of discharge) | NA | Stroke patients | To explore the effects of Orem self-care theory in community nursing of stroke patients. |
| (Li, 2020) | 2020 | China | Zhumadian, Henan Province | Prospective RCT | NR | Stroke patients (convalescent period) | To analyze the effects of hospital-community-family connection continuous nursing in patients with stroke. |
| (He et al., 2019) | 2019 | China | Shenzhen | Prospective RCT | NR | Stroke patients (limb dysfunction) | To explore the effects of community and family rehabilitation nursing model on improving the psychological status of stroke patients |
| (Hu et al., 2016) | 2016 | China | Shanghai | Quasi-experimental study (grouping by different communities) | NA | Stroke patients with hemiplegia | To explore the intervention effects of community rehabilitation model for stroke based on regional medical alliance. |
| (Zhang et al., 2022) | 2022 | China | Beijing | Prospective RCT | Random number table | Stroke patients | To evaluate the effects of community health service model for the elderly stroke patients. |
| (Chen et al., 2019) | 2019 | China | Shanghai | Prospective RCT | Random number table | Patients with post-stroke depression | To evaluate the effects of family doctor team intervention on depression and activities of daily living in community patients with PSD. |
| (Wu, 2017) | 2017 | China | Ningbo, Zhejiang Province | Prospective RCT | NR | Stroke patients (limb dysfunction) | To explore the effects of community rehabilitation treatment on improving the activity of daily living and quality of life among patients with stroke. |
| (Wu, 2020) | 2020 | China | Suzhou, Jiangsu Province | Quasi-experimental study (before and after control) | NA | Ischemic stroke patients with sequelae | To clarify the value of community rehabilitation nursing management among cerebral infarction patients. |
| (Xiao, 2013) | 2013 | China | Guangzhou, Guangdong Province | Prospective RCT | NR | Stroke patients | To explore the effects of community rehabilitation treatment on the recovery and quality of life of stroke patients |
| (Cao et al., 2016) | 2016 | China | Jiangmen, Guangdong Province | Prospective RCT | NR | Stroke patients with hemiplegia | To explore the effective home rehabilitation model for patients after stroke in rural area of Enping city on their activities of daily living, quality of life, anxiety and depression． |
| (Liu et al., 2021) | 2021 | China | Beijing | Prospective RCT | Random number table | Stroke patients (limb dysfunction) | To explore the effects of home-based telerehabilitation guidance on physical function and activities of daily living for stroke patients. |
| (Zhang et al., 2010) | 2010 | China | Jinhua, Zhejiang Province | Quasi-experimental study (before and after control) | NA | Senile stroke patients (convalescent period) | To explore the effects of home care knowledge training in community elderly patients with stroke. |
| (Mao et al., 2018) | 2018 | China | Shanghai | Prospective RCT | Computerized random number generator | Stroke patients (limb dysfunction) | To explore the effects of community rehabilitation guidance based on family doctor for stroke patients. |
| (Yu et al., 2022) | 2022 | China | Weihai, Shandong Province | Quasi-experimental study (grouping by different communities) | NA | Stroke patients | To explore the effects of applying information-based medical collaborative management and family cooperative management in stroke patients. |
| (Li et al., 2010) | 2010 | China | Nanning, Guangxi Zhuang Autonomous Region | Prospective RCT | NR | Urban stroke patients with limb dysfunction | To explore the effects of community family rehabilitation nursing on the psychological status of patients with stroke in urban community. |
| (Li et al., 2019) | 2019 | China | Zhengzhou, Henan Province | Quasi-experimental study (admission order grouping) | NA | Ischemic stroke patients with hemiplegia | To explore the effects of hospital-community-family rehabilitation nursing model on compliance behavior and daily living ability of patients with cerebral infarction and hemiplegia. |
| (Huang and Zhao, 2011) | 2011 | China | Beijing | Prospective RCT | Random number table | Stroke patients | To explore the effects of community nursing process management on the quality of community nursing for stroke patients. |
| (Xue et al., 2016) | 2017 | China | Heyuan, Guangdong Province | Prospective RCT | NR | Ischemic stroke patients (convalescent period, partial limb dysfunction) | To explore the effects of community team service on the rehabilitation of patients with cerebral infarction. |
| (Meng et al., 2022) | 2022 | China | Hohhot, Inner Mongolia Autonomous Region | Prospective RCT | NR | Stroke patients with hemiplegia (convalescent period) | To explore the effects of different stages of stroke hemiplegia rehabilitation training combined with remote scientific rehabilitation guidance and education on the patients' limb motor function and daily living ability. |
| (Xia and Zhu, 2004) | 2004 | China | Dalian, Liaoning Province | Prospective RCT | NR | Stroke patients with hemiplegia (convalescent period) | To investigate the effects of community rehabilitation on motor function and activity of daily living of convalescent stroke patient. |
| (Xue et al., 2016) | 2016 | China | Shanghai | Prospective RCT | Random number table | Stroke patients (limb dysfunction) | NR |
| (Yang et al., 2019) | 2019 | China | Beijing | Prospective RCT | NR | Ischemic stroke patients (motor dysfunction) | To explore the effects and advantages of community remote rehabilitation system in the treatment of patients with cerebral infarction. |
| (Liao et al., 2019) | 2019 | China | Jiaxing, Zhejiang Province | Prospective RCT | NR | Ischemic stroke patients (convalescent period, limb dysfunction) | To investigate the effects of the community stroke rehabilitation model on neurological rehabilitation, cerebral hemodynamics and cerebral vascular reserve in patients with cerebral infarction. |
| (Wang, 2005) | 2005 | China | Shenyang, Liaoning Province | Prospective RCT | NR | Stroke patients (convalescent period) | To comprehend the role of neurologists in the community intervention for the functional prognosis of patients with stroke. |
| (Li, 2017) | 2017 | China | Jinan, Shandong Province | Prospective RCT | NR | Stroke patients | To explore the effects of community rehabilitation treatment on the activity ability of stroke patients. |
| (Li et al., 2014) | 2014 | China | Harbin, Heilongjiang Province | Prospective RCT | Block randomization | Stroke patients (limb dysfunction) | To observe the application effects of community rehabilitation pathway in family rehabilitation of stroke patients, and to explore the standardized nursing model of family rehabilitation of stroke patients. |
| (Zhu et al., 2014) | 2014 | China | Foshan, Guangdong Province | Prospective RCT | NR | Stroke patients (convalescent period) | To observe the effects of family bed in community rehabilitation of stroke patients. |
| (Cao et al., 2013) | 2013 | China | Dongguan, Guangdong Province | Quasi-experimental study (before and after control) | NA | Stroke patients | To observe the effects of group therapy in social rehabilitation training on stroke patient. |
| (Liu, 2015) | 2014 | China | Wuhan, Hubei Province | Prospective RCT | NR | Stroke patients with hemiplegia (limb dysfunction) | To explore the influence of the family as the unit self-management mode on the rehabilitation of patients with cerebral apoplexy. |
| (Zhang et al., 2015) | 2015 | China | Shanghai | Prospective RCT | Random number table | Stroke patients (limb dysfunction) | To explore the effects of the community stroker rehabilitation unit on rehabilitation of the patients with the stroke. |
| (Chen et al., 2020) | 2020 | China | Taizhou, Zhejiang Province | Prospective RCT | Random number table | Senile stroke patients (convalescent period) | To observe the application effects of functional gait training in community realistic environment among elderly stroke patients. |
| (Lv, 2020) | 2020 | China | Dongying, Shandong Province | Prospective RCT | Random number table | Senile stroke patients (convalescent period) | To analyze the application effects of home care knowledge training in community elderly patients with stroke. |
| (Xue et al., 2015) | 2015 | China | Shanghai | Prospective RCT | Random number table | Stroke patients (limb dysfunction) | To explore the impact of the family rehabilitation on the life quality, anxiety and depression of patients with the stroke under the community team mode. |
| (Chen et al., 2018) | 2018 | China | Zhaoqing, Guangdong Province | Prospective RCT | NR | Stroke patients with hemiplegia | To explore the effects of hospital-community-family continuous rehabilitation nursing intervention on physical function and mental state of stroke patients |
| (Jiao et al., 2013) | 2013 | China | Beijing | Prospective RCT | Random number table | Stroke patients with hemiplegia | To explore the effects of hospital-guided community-based comprehensive rehabilitation on limb motor function, balance function and activities of daily living in stroke patients with hemiplegia. |
| (Liang et al., 2016) | 2016 | China | Nanning, Guangxi Zhuang Autonomous Region | Prospective RCT | NR | Patients with post-stroke depression | To investigate the effects of community psychological intervention based on five-element theory on neural function in patients with PSD. |
| (Xu et al., 2020) | 2020 | China | Dalian, Liaoning Province | Prospective RCT | Random number table | Acute ischemic stroke patients with hemiplegia | To explore the application effects of hospital-community-family rehabilitation nursing model in stroke patients with hemiplegia. |
| (Qin et al., 2011) | 2011 | China | Nanning, Guangxi Zhuang Autonomous Region | Prospective RCT | Drawing lots | Stroke patients (motor dysfunction) | To explore the effective rehabilitation nursing program for patients with stroke in community |
| (Gao et al., 2014) | 2014 | China | Wuhan, Hubei Province | Prospective RCT | NR | Stroke patients with hemiplegia | To investigate the effects of core stability training on trunk control ability, balance function, motor function, walking ability, gait and activities of daily living in community stroke patients with hemiplegia. |
| (Liu et al., 2022) | 2022 | China | Zhengzhou, Henan Province | Prospective RCT | Random number table | Senile stroke patients | To explore the implementation of collaborative rehabilitation management model for community elderly patients with post-stroke urinary incontinence based on family doctor team. |
| (Wang, 2016) | 2016 | China | Guangzhou, Guangdong Province | Prospective RCT | NR | Stroke patients | To analyze the effects of appropriate community-based rehabilitation intervention on motor function of stroke patients |
| (Wang et al., 2020) | 2020 | China | Haikou, Hainan Province | Prospective RCT | Random number table | Stroke patients | To observe the effects of family doctor contracted service on self-perceived burden and psychological state of stroke patients in community. |
| (Meng and Wang, 2015) | 2015 | China | Nanjing, Jiangsu Province | Prospective RCT | Random number table | Stroke patients (convalescent period) | To explore the effects of transitional care on self-care ability of stroke patients returning to community. |
| (Moore et al., 2015) | 2015 | UK | Newcastle upon Tyne | A single-center, single-blind, parallel, randomized controlled trial | Computerized random number generator | Senile stroke patients | To explore the short-term metabolic, brain, cognitive, and functional effects of exercise following stroke. |
| (Yu et al., 2009) | 2009 | China | Shanghai | A single-blind, randomized control design | Throwing coins randomly | Stroke patients | To evaluate the effects of community-based rehabilitation therapy on neurological function deficit among stroke patients. |
| (Daviet et al., 2023) | 2022 | France | Limoges, Haute Vienne department | Retrospective cohort study (without control group) | NA | Stroke patients | To examine whether there is an improvement in the social participation of patients who received a rehabilitation program provided by community stroke rehabilitation teams. The secondary objectives were to show if there is an improvement in the patients’ quality of life and a reduction in the caregiver burden. |
| (Eng et al., 2003) | 2003 | Canada | Vancouver, BC | A single group, repeated measures design | NA | Stroke patients with hemiplegia | to evaluate the physical and psychosocial effects of an 8-week community-based functional exercise program in a group of individuals with chronic stroke. |
| (Lee et al., 2017) | 2017 | USA | Chicago, IL | A quasi-experimental design with a control group and pre-/post-test design but without randomization | NA | Stroke patients | To evaluate the feasibility and effectiveness of the Improving Participation After Stroke Self-management program – Rehab version (IPASS-R) in a day rehabilitation setting. |
| (Park and Lee, 2016) | 2016 | Korea | Gyeongsangnam-do | A single group, repeated measures design | NA | Senile stroke patients | To examine the effects of community-based rehabilitation program in chronic stroke patients. |
| (Bishop et al., 2014) | 2014 | USA | Providence, Rhode Island | Prospective RCT | Urn randomization | Stroke patients and their caregivers | To preliminarily test the efficacy of a telephone intervention, Family Intervention: Telephone Tracking, designed to assist stroke survivors and their primary caregivers during the ﬁrst 6 months after stroke. |

Note. NR: none reported; NA: not applicable.

Bishop, D., Miller, I., Weiner, D., Guilmette, T., Mukand, J., Feldmann, E., Keitner, G., Springate, B., 2014. Family Intervention: Telephone Tracking (FITT): a pilot stroke outcome study. Topics in stroke rehabilitation 21 Suppl 1, S63-74.

Cai, L.N., Shi, J.L., Chen, X.L., 2021. Effectiveness of "hospital-community-family" extended care in elderly patients with ischemic stroke. Nursing of Integrated Traditional Chinese and Western Medicine 7 (8), 136-138.

Cao, Q.R., Feng, S.W., Huang, S.Y., Li, X.Y., Lin, X.Y., Tan, Y.M., Xu, C.L., 2016. Study of home rehabilitation model and effectiveness analysis on stroke patients in rural area. Chinese Journal of Rehabilitation Theory and Practice 31 (3), 190-192.

Cao, Q.Y., Zhong, H.M., Liu, Q.E., Zhang, H.M., Liao, Y.Y., 2013. Application of group therapy in social rehabilitation training on stroke patients. China Medicine and Pharmacy 3 (11), 47-48.

Chen, L., 2021. The effect of hospital-community-family rehabilitation nursing model on the psychological state and daily living ability of patients with cerebral infarction. Heilongjiang Journal of Traditional Chinese Medicine 50 (3), 232-233.

Chen, Q., Shi, H.Q., Wu, Z.H., Zhi, B.H., Shi, M.F., 2018. The role of hospital-community-family extended rehabilitation nursing intervention in improving physical function and psychological status of stroke patients. Chronic Pathematology Journal 19 (2), 228-230.

Chen, R.X., Guan, Z.J., Lu, Y.X., Huang, K.F., Deng, L.M., Chen, S.Y., 2012. Impact of a combined hospital-community-home care model on the quality of life of depressed patients after stroke. International Medicine & Health Guidance News 18 (15), 2166-2168.

Chen, R.X., Guan, Z.J., Wu, Y.E., Fang, Y.G., Wei, Y.L., Fang, Y.H., 2012. Effects of care provided by hospital, community and family on quality of life of stroke patients. Journal of Nursing Science 27 (8), 82-84.

Chen, S., 2018. Effect of the hospital-community linkage nursing management mode on the rehabilitation and quality of life in patients with stroke. Journal of Bengbu Medical College 43 (1), 110-113.

Chen, W., Jiang, B., Zhu, H.X., Yang, Z.J., Xu, G.Z., Peng, L., Wei, J., 2019. The effect of rehabilitation of family doctor team intervention on community post-stroke depression patients. Shanghai Medical & Pharmaceutical Journal 40 (10), 56-58.

Chen, W.P., Lin, D., Lu, W., 2020. A study on the application of functional gait training in a community-based realistic environment in elderly patients in the rehabilitation period after stroke. Journal of Nursing and Rehabilitation 19 (5), 65-68.

Chi, X.Q., 2009. Community care of nine cases of hemiplegic stroke with motor function rehabilitation. Chinese Journal of Rural Medicine and Pharmacy 16 (3).

Dan, S.C., Gao, L., Ge, X.H., He, Q., 2015. Effect of Community Rehabilitation on activity of daily living in stroke patients. Heilongjiang Medicine Journal 28 (4), 887-889.

Daviet, J.C., Compagnat, M., Bonne, G., Maud, L., Bernikier, D., Salle, J.Y., 2023. Individualized home-based rehabilitation after stroke in France: a pragmatic study of a community stroke rehabilitation team. Canadian Journal of Neurological Sciences 50 (3), 405-410.

Eng, J.J., Chu, K.S., Kim, C.M., Dawson, A.S., Carswell, A., Hepburn, K.E., 2003. A community-based group exercise program for persons with chronic stroke. Medicine and Science in Sports and Exercise 35 (8), 1271-1278.

Fu, X.M., Jin, S.J., Zeng, X.L., Chen, Y., 2019. Effect of home pension system under medical-nursing combined model on quality of life in elderly patients with stroke. Hainan Medical Journal 30 (10), 1352-1355.

Gao, C.H., Huang, X.L., Zhang, W., Cai, J.H., Liu, Y.L., Wang, W., 2014. The effects of core stability training on stroke patients' motor function. Stroke and Nervous Diseases 21 (4), 207-211.

Gao, S.F., Sun, P.Y., Jiao, L.Q., 2013. Effects of different community rehabilitation models on daily living ability and neuropsychology of patients with cerebral infarction. Chinese Journal of Integrative Medicine on Cardio-/Cerebrovascular Disease 11 (7), 826-827.

He, M.L., Xie, Y.H., Wang, W.H., Li, H.S., 2019. The effect of community-based home rehabilitation care model on improving the psychological status of stroke patients. Journal of Qilu Nursing 25 (3), 105-107.

He, Y., 2015. Exploring the effectiveness of community-based rehabilitation guidance in stroke. Scientific & Technical Information of Gansu 44 (6), 116-117.

Hu, S.H., Ling, Q., Xu, J., Jiang, L.J., Lu, Y., Su, N., Hu, J.Q., Zhang, X.F., Shen, M.H., Li, R.Y., 2016. Intervention effect of community rehabilitation model in stroke patients based on regional medical association. Chinese General Practice 19 (22), 2729-2733.

Hu, X.X., Li, H., 2016. Effect of community care and family members' participation on rehabilitation of patients with cerebral infarction. Modern Clinical Nursing 15 (5), 26-30.

Huang, C.X., Zhao, S.H., 2011. The impact of process management on quality control of community care for stroke patients. Chinese General Practice 14 (35), 4028-4031.

Huang, W.L., He, Y.J., 2012. Influence of quantitative nursing intervention on rehabllitation of community stroke patients. Modern Hospitals 12 (7), 151-153.

Jiang, M.H., Qin, B., Chen, Q.G., 2010. The effect of community rehabilitation on the quality of life of homebound stroke patients with hemiplegia. Nursing Practice and Research 7 (13), 117-119.

Jiao, L.Q., Gao, S.F., Sun, P.Y., 2013. A clinical study of comprehensive community-based rehabilitation for stroke hemiparesis directed by a general hospital. Chinese Journal of Integrative Medicine on Cardio-/Cerebrovascular Disease 11 (10), 1218-1219.

Lan, Q., Ji, M.L., Chen, L.Q., 2008. A study of the effectiveness of stroke home rehabilitation team interventions for patients in the community. Shanghai Nursing 8 (4), 34-36.

Lee, D., Fischer, H., Zera, S., Robertson, R., Hammel, J., 2017. Examining a participation-focused stroke self-management intervention in a day rehabilitation setting: a quasi-experimental pilot study. Topics in Stroke Rehabilitation 24 (8), 601-607.

Li, G.Z., 2014. Effectiveness of Orem's self-care theory in the community care of stroke patients. Chinese Journal of Trauma and Disability Medicine 22 (6), 273-274.

Li, H., 2020. Hospital-community-family interface continuity of care in patients recovering from stroke. Shanxi Medical Journal 49 (13), 1748-1750.

Li, L., Li, S.W., Zhao, H., Pan, H., Zhang, L., Han, S., Zhao, L., Wu, G.S., Mao, J.F., Li, Y., 2014. Effect evaluation of the application of community rehabilitation pathway in home rehabilitation. Heilongjiang Medical Journal 38 (11), 1331-1334.

Li, L., Yue, P., Zhang, Y., 2019. The effect of hospital-community-family rehabilitation nursing model on medical compliance behavior and daily living ability of hemiplegic patients with cerebral infarction. Henan Medical Research 28 (5), 913-915.

Li, X.M., 2017. Effectiveness of community-based rehabilitation therapy in improving activities of daily living of stroke patients. Biped and Health 26 (24), 59-60.

Li, X.P., Wang, L., Lan, Y.L., Huang, W.D., Zhang, Q., 2010. Impact of community home-based rehabilitation nursing on psychological state of cerebral apoplexy patients in urban communities. Chinese Nursing Researsh 24 (3), 838-839.

Li, X.P., Wang, L., Zhang, Q., Huang, W.D., Lai, G.F., 2011. Effectiveness study of nursing by rehabilitation collaboration network among stroke family in urban community. Journal of Nurses Training 26 (9), 773-776.

Liang, N., Wang, Z.K., Zhang, Z.W., Chen, D., Lu, X.J., Song, X., Chen, R.Q., Hu, Y.Y., He, Q.C., Qin, Q.Q., 2016. Influence of community psychological intervention based on five-element theory on neural function in patients with post-stroke depression. Internal Medicine 11 (2), 174-176.

Liao, Q.H., Wang, F., Xu, W.W., Zhi, J.F., Chen, S.L., Li, J., 2019. Impact of a community-based stroke rehabilitation model on neurological rehabilitation in recovery from cerebral infarction. Zhejiang Clinical Medical Journal 21 (2), 204-206.

Liu, C.F., Zhang, J., Li, X.X., Chen, Y.R., Zhang, Z.X., Sun, X.Y., Miao, Y.Z., Wang, L.L., 2022. Effect of collaborative rehabilitation intervention on functional recovery and quality of life in elderly stroke patients. Chinese Journal of Practical Nervous Diseases 25 (2), 197-201.

Liu, H.L., Zhou, B., Zhao, Z., Yang, Y., Lv, X.Q., Wang, Y., Yu, T., 2021. Home-based telerehabilitation guidance for stroke patients. Chinese Journal of Rehabilitation Theory and Practice 27 (7), 807-811.

Liu, S.S., 2015. Take the family as the influence of self-management mode on rehabilitation of patients with cerebral apoplexy. China Health Industry 12 (8), 142-144.

Liu, Y., Liu, J.J., Jin, J.P., 2019. The efficacy of the "three-society linkage" model for community-based rehabilitation of stroke patients. Chinese Journal of Gerontology 39 (5), 1051-1053.

Lv, Q.F., 2020. Analysis of the effect of home care knowledge training in community-based elderly people recovering from stroke. Reflexology and Rehabilitation Medicine (9), 167-168.

Mao, J.B., Hu, H.J., Zhang, J.M., 2018. Influence of community family doctor as the center on rehabilitation of functional recovery of patients with stroke. Shanghai Medical & Pharmaceutical Journal 39 (8), 60-62.

Meng, F.Y., Wang, Y., 2015. Influence of continuity nursing on self-care ability of cerebral apoplexy patients in community during rehabilitation. Chinese Nursing Researsh 29 (6), 2215-2218.

Meng, Y.Q., Liu, G.J., Fan, Z.C., Qi, H., Bao, S.R.L., Zhu, R.X., 2022. Application of remote scientific rehabilitation guidance and education in hemiplegia rehabilitation. International Medicine & Health Guidance News 28 (15), 2127-2131.

Moore, S.A., Hallsworth, K., Jakovljevic, D.G., Blamire, A.M., He, J.B., Ford, G.A., Rochester, L., Trenell, M.I., 2015. Effects of community exercise therapy on metabolic, brain, physical, and cognitive function following stroke: a randomized controlled pilot trial. Neurorehabilitation and Neural Repair 29 (7), 623-635.

Park, Y.-J., Lee, C.-Y., 2016. Effects of community-based rehabilitation program on activities of daily living and cognition in elderly chronic stroke survivors. Journal of physical therapy science 28 (11), 3264-3266.

Qin, Y., Li, X.P., Wang, L., 2011. Observation on the effect of community rehabilitation nursing care for patients with post-stroke sequelae. Journal of Nursing (China) 18 (6), 63-65.

Rao, R., Ye, D., Hu, J., 2014. A study of out-of-hospital acceptance of continuity of care services for patients recovering from stroke. Chinese Journal of Rehabilitation 29 (6), 453-454.

Shi, S.X., Xu, W.W., Liu, X., Huang, J., Guo, Y., Fu, L., Jin, H., 2022. Study on the efficacy of community-led home rehabilitation model on stroke patients under the background of Internet plus. Journal of Modern Medicine & Health 38 (17), 2904-2907.

Wang, H., Wu, H., Wang, S.P., 2020. The effects of contracted services of family physicians on self-perceived burden and psychological status of stroke patients in a community. Chinese Nursing Management 20 (2), 276-281.

Wang, J.J., Xie, P., Bai, J.X., Cai, G.L., 2022. Impact of hospital-community-home continuity of care on stroke patients with non-dementia cognitive impairment. Today Nurse 29 (2), 84-87.

Wang, L., 2005. Neurologist participates in the community intervention for the functional prognosis of convalescent patients with stroke. Chinese Journal of Clinical Rehabilitation 9 (17), 4-5.

Wang, M.H., 2016. Analysis of the effect of implementing appropriate community-based rehabilitation interventions on motor function in 60 stroke patients. Chinese Journal of Trauma and Disability Medicine 24 (15), 55-56.

Wei, X.P., Yu, L.M., Hu, W., 2009. Observation of curative effects on treatment of patients with limb disability sequela after stroke in community health service. Journal of Neurology and Neurorehabilitation 6 (3), 191-193.

Wu, L., 2020. Effective application of community rehabilitation nursing in the rehabilitation management of patients with sequelae of cerebral infarction. Yi Shou Bao Dian (10), 0058-0058.

Wu, L.B., 2017. An analysis of the effects of community-based rehabilitation for stroke patients. Contemporary Medicine Symposium 15 (10), 59-60.

Wu, M.H., Zhu, C.P., Xu, X.F., Lu, A.M., Chu, H.F., 2017. Effect of home-based rehabilitation nursing on the ability of activities of daily living in the rural patients with stroke. Shanghai Medical & Pharmaceutical Journal 38 (22), 60-62.

Xia, W.M., Zhu, P., 2004. The effect of community rehabilitation on motor function of post-stroke patients. Clinical Journal of Medical Officer 32 (5), 97-98.

Xiao, X.F., 2013. Effects of community rehabilitati0n on recovery in patients with stroke and quality of life. China Modern Medicine 20 (26), 162-163.

Xu, H., Xin, Y.Y., Yin, H.X., Wang, Y.L., Wang, X.F., Tan, Y.J., 2020. Effect of hospital-community-family rehabilitation nursing model on hemiplegic patients with acute cerebral infarction. China Modern Medicine 27 (5), 239-242.

Xue, B., Gu, W.Q., Tang, Z.Y., Liu, T.L., Zhao, S., Qin, D., 2015. Study of the impact of the family rehabilitation on the life quality, anxiety and depression of the patients with the stroke under the community team mode. Shanghai Medical & Pharmaceutical Journal 36 (20), 60-62.

Xue, B., Tang, Z.Y., Liu, T.L., Zhao, S., Qin, D., Gu, W.Q., 2016. Effects of home rehabilitation in a community team model on physical function and activities of daily living in stroke patients. Shanxi Medical Journal 45 (6), 727-729.

Yang, Y., Ma, Y.H., Zhao, Z., Zhou, B., Liu, H.L., 2019. Study on the advantages and effects of applying community-based tele-rehabilitation for patients with cerebral infarction. World Latest Medicine Information 19 (24), 84-89.

Yu, J., Hu, Y., Wu, Y., Chen, W., Zhu, Y., Cui, X., Lu, W., Qi, Q., Qu, P., Shen, X., 2009. The effects of community-based rehabilitation on stroke patients in China: a single-blind, randomized controlled multicentre trial. Clinical Rehabilitation 23 (5), 408-417.

Yu, X.Y., Sang, S.H., Chi, J., Du, X., Ren, Q.H., Zhang, L., 2022. Application of information-based medical collaborative management and family cooperative management in stroke patients taking rehabilitation at home. International Medicine & Health Guidance News 28 (11), 1524-1529.

Zhang, W.F., Chen, L.N., 2018. Effects of mobile phone App based extended nursing care on the stroke patients in community. China Journal of Modern Nursing 24 (2), 190-195.

Zhang, X.C., Li, S.H., Qian, Y.F., Wang, L.Q., Yu, B., 2010. Observation on the effect of home care knowledge training for elderly people recovering from stroke in the community. Journal of Nursing (China) 17 (11), 65-67.

Zhang, X.M., Zhu, W.H., Jiang, Q.K., Gan, L.F., 2020. Efficiency of community and family-based rehabilitation strategy in the rehabilitation of post-stroke depression patients. Chinese journal of Clinical Medicine 27 (4), 657-661.

Zhang, X.Q., Wang, C., Bi, Z.Z., Gu, Y.M., Jin, L.J., Jin, R.X., Chen, L.B., Liu, Y.L., 2015. Establishment of the community stroke rehabilitation unit and evaluation of its operational result. Shanghai Medical & Pharmaceutical Journal 36 (4), 57-59.

Zhang, X.Q., Wang, C., Bi, Z.Z., Gu, Y.M., Liu, Y.L., Jin, L.J., Jin, R.X., Chen, L.B., 2015. Effect of the community-based rehabilitation unit on stroke: a prospective randomized, control study. Shanghai Medical & Pharmaceutical Journal 36 (12), 47-50.

Zhang, Y., Wang, L., Liu, Y., Gao, Y., Qian, F., Wang, Y.Q., 2022. Application effects of community elderly health service model in home health management of stroke patients. Chinese Nursing Management 22 (3), 334-338.

Zhu, J.X., Zhou, F.Y., Deng, H.D., Li, Y.L., 2014. The role of home beds in community-based rehabilitation of patients recovering from stroke. Chinese Journal for Clinicians 42 (6), 41-42.
