# Supplementary material for: Community-based rehabilitation services implemented by multidisciplinary teams among adults with stroke: a scoping review with a focus on Chinese experience
Source: BMC Public Health. 2024 Mar 7;24:740. doi: 10.1186/s12889-024-18218-1 (PMC10921794; doi:10.1186/s12889-024-18218-1)
Supplement: Supplementary file 3 — Supplementary Material 3 [file 12889_2024_18218_MOESM3_ESM.docx]

Table S2 Characteristics of the sample population for included studies (N=74)

| Authors & year of publication | Sample size N=, (Control) | Age (M ± SD) | | Male (n=, %) or Male/Female | | Ischemic/Hemorrhagic (Ischemic, %) | | Chronic disease (% reported n=) | |
| --- | --- | --- | --- | --- | --- | --- | --- | --- | --- |
|  |  | Intervention | Control | Intervention | Control | Intervention | Control | Intervention | Control |
| (Wang et al., 2022) | 112  (Control= 56) | (64.84±4.96) years | (66.25±5.30) years | 33(58.9%) | 36(64.3%) | 37/19 | 40/16 | hypertension/hyperlipidemia/diabetes/coronary heart disease=32/15/17/9 | hypertension/hyperlipidemia/diabetes/coronary heart disease=30/19/11/6 |
| (Zhang and Chen, 2018) | 68  (Control= 34) | (66.90±5.70) years | (67.00±5.20) years | 18(52.9%) | 19(55.9%) | 27/7 | 29/5 | NR | NR |
| (Lan et al., 2008) | 46  (Control= 20) | (66.00±9.60) years | (70.90±9.00) years | 18(69.2%) | 10(50.0%) | NR | NR | NR | NR |
| (Chen, 2021) | 86  (Control= 43) | (52.43±3.01) years | (52.56±3.02) years | 23(53.5%) | 22(51.2%) | 43/0 | 43/0 | NR | NR |
| (Zhang et al., 2015) | 136  (Control= 50) | (56.7±5.4) years | | NR | NR | 84/52 | | NR | NR |
| (Jiang et al., 2010) | 36  (Control= 18) | (64.06±4.50) years | (60.78±4.09) years | 12(66.7%) | 13(72.2%) | 9/9 | 8/10 | NR | NR |
| (Chen et al., 2012) | 72  (Control= 36) | (63.60±10.10) years | (62.80±10.80) years | 23(63.9%) | 25(69.4%) | NR | NR | NR | NR |
| (Chen, 2018) | 120  (Control= 60) | 48~78 years | 48~75 years | 32(53.3%) | 30(50.0%) | 22/38 | 25/35 | NR | NR |
| (Cai et al., 2021) | 120  (Control= 60) | (67.77±4.83) years | (67.23±5.48) years | 36(60.0%) | 32(53.3%) | 60/0 | 60/0 | hypertension/diabetes/coronary heart disease=45/23/13 | hypertension/diabetes/coronary heart disease=38/19/9 |
| (Fu et al., 2019) | 127  (Control= 64) | (72.20±12.17) years | (71.78±13.24) years | 35(55.6%) | 35(54.7%) | 41/22 | 43/21 | NR | NR |
| (Chi, 2009) | 9 | 44~70 years  (Mean age =57.1) | NA | 6(66.7%) | NA | NR | NA | NR | NA |
| (Wu et al., 2017) | 84  (Control= 42) | (67.71±6.61) years | (70.60±6.23) years | 23(54.8%) | 21(50.0%) | 31/11 | 32/10 | NR | NR |
| (Chen et al., 2012) | 100  (Control= 50) | (65.60±11.10) years | (64.80±10.80) years | 32(64.0 %) | 33(66.0%) | 36/14 | 33/17 | NR | NR |
| (Zhang et al., 2020) | 63  (Control= 31) | (72.50±8.60) years | (72.80±9.40) years | 13(40.6%) | 13(41.9%) | 26/6 | 21/10 | hypertension/hyperlipidemia/diabetes/coronary heart disease=22/19/7/12 | hypertension/hyperlipidemia/diabetes/coronary heart disease=18/20/9/12 |
| (Dan et al., 2015) | 60  (Control= 30) | NR | NR | NR | NR | NR | NR | NR | NR |
| (He, 2015) | 86  (Control= 29) | (59.30±13.30) years | (61.10±13.30) years | 31(54.4%) | 11(37.9%) | NR | NR | NR | NR |
| (Huang and He, 2012) | 84  (Control= 42) | (64.62±7.98) years | (64.45±7.98) years | 26(61.9%) | 25(59.5%) | NR | NR | NR | NR |
| (Rao et al., 2014) | 60 | (60.54±6.80) years | NA | 49(81.7%) | NA | NR | NA | NR | NA |
| (Wei et al., 2009) | 66  (Control= 34) | (59.31±8.03) years | (56.94±8.59) years | 17(53.1%) | 18(52.9%) | 18/14 | 19/15 | NR | NR |
| (Gao et al., 2013) | 100  (Control= 50) | (61.86±9.03) years | (62.53±8.45) years | 33(66.0%) | 32(64.0%) | 42/8 | 40/10 | hypertension/diabetes=25/13 | hypertension/diabetes=27/12 |
| (Shi et al., 2022) | 60  (Control= 30) | (68.70±7.70) years | (70.30±8.60) years | 17(56.7%) | 14(46.7%) | NR | NR | NR | NR |
| (Liu et al., 2019) | 80  (Control= 40) | (58.30±12.80) years | (62.20±11.80) years | 28(70.0%) | 26(65.0%) | 36/4 | 34/6 | hypertension/hyperlipidemia/diabetes/coronary heart disease=36/21/31/14 | hypertension/hyperlipidemia/diabetes/coronary heart disease=35/20/31/13 |
| (Li et al., 2011) | 80  (Control= 40) | (64.57±8.69) years | (65.42±8.45) years | 23(57.5%) | 25(62.5%) | 32/8 | 30/10 | NR | NR |
| (Hu and Li, 2016) | 42 | (66.15±7.23) years | NA | 19(45.2%) | NA | 42/0 | NA | NR | NA |
| (Li, 2014) | 80  (Control= 40) | NR | NR | NR | NR | NR | NR | NR | NR |
| (Li, 2020) | 87  (Control= 42) | (56.00±5.00) years | (54.00±5.00) years | 23(51.1%) | 21(50.0%) | NR | NR | NR | NR |
| (He et al., 2019) | 56  (Control= 28) | (68.40±9.60) years | (65.40±8.70) years | 16(57.1%) | 14(50.0%) | 13/15 | 12/16 | NR | NR |
| (Hu et al., 2016) | 106  (Control= 52) | (59.30±9.60) years | (58.70±9.10) years | 36(67.9%) | 33(63.5%) | 39/14 | 40/12 | NR | NR |
| (Zhang et al., 2022) | 60  (Control= 30) | (66.53±8.09) years | (66.30±6.83) years | 21(70.0%) | 18(60.0%) | 21/9 | 20/10 | NR | NR |
| (Chen et al., 2019) | 90  (Control= 45) | (69.97±7.90) years | (70.88±7.73) years | 28(62.2%) | 26(57.8%) | 43/2 | 42/3 | NR | NR |
| (Wu, 2017) | 68  (Control= 34) | (68.17±12.05) years | (67.84±11.49) years | 18(52.9%) | 19(55.9%) | 24/10 | 25/9 | NR | NR |
| (Wu, 2020) | 13 | (63.40±0.50) years | NA | 9(47.4%) | NA | 13/0 | NA | NR | NA |
| (Xiao, 2013) | 136  (Control= 68) | (61.90±10.00) years | (63.20±11.50) years | 38(55.9%) | 36(52.9%) | NR | NR | NR | NR |
| (Cao et al., 2016) | 100  (Control= 48) | (60.80±14.40) years | (67.20±13.60) years | 34(65.4%) | 29(60.4%) | 32/16 | 29/21 | NR | NR |
| (Liu et al., 2021) | 222  (Control= 114) | (65.70±13.06) years | (63.79±11.14) years | 52(48.1%) | 68(59.6%) | 50/58 | 66/48 | NR | NR |
| (Zhang et al., 2010) | 21 | (78.78±4.41) years | NA | 14(66.7%) | NA | 12/9 | NA | diabetes/hypertension/hyperlipidemia/xerosis/cataract=13/15/12/1/1 | NA |
| (Mao et al., 2018) | 190  (Control= 95) | (72.63±13.56) years | (66.83±11.68) years | 59(62.1%) | 62(65.3%) | NR | NR | NR | NR |
| (Yu et al., 2022) | 60  (Control= 30) | (64.21±3.54) years | (63.72±3.97) years | 18(60.0%) | 19(63.3%) | NR | NR | hypertension/ diabetes/ hyperlipidemia=21/11/13 | hypertension/ diabetes/ hyperlipidemia=20/10/15 |
| (Li et al., 2010) | 69  (Control= 25) | (65.73±8.35) years | (65.11±8.71) years | 20(58.8%) | 22(62.9%) | 27/7 | 25/10 | NR | NR |
| (Li et al., 2019) | 116  (Control= 58) | (58.19±7.81) years | (57.70±7.64) years | 33(56.9%） | 32(55.2%) | 58/0 | 58/0 | NR | NR |
| (Huang and Zhao, 2011) | 101 (Control= 50) | age<55-n=11,55~71years-n=24, >71years-n=16 | age<55-n=11,55~71years-n=23, >71years-n=16 | 33(64.7%) | 33(66.0%) | NR | NR | NR | NR |
| (Xue et al., 2016) | 120  (Control= 60) | (49.38±3.04) years | (51.22±2.37) years | 36(60.0%) | 33(55.0%) | 60/0 | 60/0 | NR | NR |
| (Meng et al., 2022) | 64  (Control= 32) | (60.47±7.82) years | (59.78±7.62) years | 20(62.5%) | 19(59.4%) | NR | NR | NR | NR |
| (Xia and Zhu, 2004) | 50  (Control= 24) | (55.60±7.20) years | (53.60±8.10) years | 17(65.4%) | 16(66.7%) | 18/8 | 19/5 | NR | NR |
| (Xue et al., 2016) | 102  (Control= 50) | 45~50 years-n=2, 51~60 years-n=4, 61~70 years-n=16, 71~80 years-n=18,  >80 years-n=12 | 45~50 years-n=3, 51~60 years-n=5, 61~70 years-n=18, 71~80 years-n=18,  >80 years-n=6 | 26(50.0%) | 24(48.0%) | 48/4 | 43/7 | NR | NR |
| (Yang et al., 2019) | 90  (Control= 45) | Mean age=63.5 years age range=52~63 years | Mean age=62.9 years,  age range=50~65 years | 30(66.7%) | 29(64.4%) | 45/0 | 45/0 | diabetes/ hypertension=14/33 | diabetes/ hypertension=15/30 |
| (Liao et al., 2019) | 80  (Control= 40) | (61.30±7.40) years | (60.2±6.8) years | 27(67.5%) | 24(60.0%) | 40/0 | 40/0 | NR | NR |
| (Wang, 2005) | 198  (Control= 52) | (63.24±7.35) years | (60.94±9.87) years | 79(54.1%) | 26(50.0%) | NR | NR | NR | NR |
| (Li, 2017) | 91  (Control= 45) | (67.20±3.80) years | (67.50±3.40) years | 25(54.3%) | 21(46.7%) | NR | NR | NR | NR |
| (Li et al., 2014) | 60 | (56.0±7.0) years | | 36/24 | | 34/26 | | NR | NR |
| (Zhu et al., 2014) | 50  (Control= 25) | NR | NR | 27/23 | | 24/26 | | NR | NR |
| (Cao et al., 2013) | 48 | (62.5±11.8) years | NA | 40(83.3%) | NA | 13/35 | NA | NR | |
| (Liu, 2015) | 100  (Control= 50) | (62.1±5.4) years | | 61/39 | | 59/41 | | NR | NR |
| (Zhang et al., 2015) | 86  (Control= 43) | (55.75±4.95) years | | 51/35 | | 34/52 | | NR | NR |
| (Chen et al., 2020) | 120  (Control= 60) | (68.57±8.33) years | (68.32±8.24) years | 36 (60.0%) | 35(58.3%) | 28/32 | 27/33 | NR | NR |
| (Lv, 2020) | 80  (Control= 40) | (78.64±4.21) years | (78.77±4.40) years | 23(57.5%) | 22(55.0%) | NR | NR | NR | NR |
| (Xue et al., 2015) | 102  (Control= 50) | NR | NR | 26(50.0%) | 24(48.0%) | NR | NR | NR | NR |
| (Chen et al., 2018) | 66  (Control= 33) | (60.7±2.6) years | (60.4±2.5) years | 18(54.5%) | 17(51.5%) | 22/11 | 23/10 | hyperglycemia/ hyperlipidemia/ hypertension =6/9/16 | hyperglycemia/hyperlipidemia/ hypertension =6/8/15 |
| (Jiao et al., 2013) | 100  (Control= 50) | (61.86±8.45) years | (62.53±8.45) years | 33(66.0%) | 32(64.0%) | 42/8 | 40/10 | diabetes/hypertension=13/25 | diabetes/hypertension=27/12 |
| (Liang et al., 2016) | 50  (Control= 25) | (56.00±14.00) years | (54.00±13.00) years | 10(40.0%) | 12(48.0%) | NR | NR | NR | NR |
| (Xu et al., 2020) | 98  (Control= 49) | (63.27±5.84) years | (63.19±5.91) years | 29(59.2%) | 28(57.1%) | 49/0 | 49/0 | NR | NR |
| (Qin et al., 2011) | 69  (Control= 35) | (65.75±8.35) years | (65.11±8.71) years | 20(58.8%) | 22(62.9%) | 27/7 | 25/10 | NR | NR |
| (Gao et al., 2014) | 100  (Control= 50) | (52.68 ± 9.27) years | (55.16±10.39) years | 28(56.0%) | 30(60.0%) | 36/14 | 35/15 | NR | NR |
| (Liu et al., 2022) | 55  (Control= 26) | (71.34±6.50) years | (72.96±6.19) years | 18(62.1%) | 14(53.8%) | 13/16 | 12/14 | NR | NR |
| (Wang, 2016) | 60  (Control= 30) | (53.18±12.34) years | | NR | NR | 49/11 | | NR | NR |
| (Wang et al., 2020) | 116  (Control= 64) | (59.23±8.37) years | (57.84±10.29) years | 33(63.5%) | 36(56.3%) | 52/0 | 64/0 | NR | NR |
| (Meng and Wang, 2015) | 97  (Control= 48) | (52.70±6.20) years | (51.00±5.80) years | 34(69.4%) | 32(66.7%) | 38/11 | 39/9 | NR | NR |
| (Moore et al., 2015) | 40  (Control= 20) | (68.00±8.00) years | (70.00±11.00) years | 18(90.0%) | 16(80.0%) | 19/1 | 18/2 | NR | NR |
| (Yu et al., 2009) | 737  (Control= 360) | 40~65, N=132;  66~85, N=245 | 40~65, N=119;  66~85, N=241 | 20(53.1%) | 19(55.3%) | 296/81 | 278/82 | hypertension/ Hyperlipidemia  296/114 | hypertension/ Hyperlipidemia  290/119 |
| (Daviet et al., 2023) | 206 | (66.3 ±12.7) years | NA | 13(66.5%) | NA | 162/44 | NA | NR | NA |
| (Eng et al., 2003) | 25 | (63.16±8.50) years | NA | 19(76.0%) | NA | 14/9, unknown=2 | NA | hypertension /obesity(BMI＞ 30) diabetes/hyperlipidemia)/clinical depression=15/5/3/5/4 | NA |
| (Lee et al., 2017) | 17 (Control= 8) | (62.00±8.00) years | (55.00±5.60) years | 1 (11.1%) | 5 (62.5%) | NR | NR | NR | NR |
| (Park and Lee, 2016) | 11 | 65~74 8 (72.8%) 75~84 2 (18.2%)  85 and above 1 (9.0%) | NA | 4(36.4%) | NA | 7/4 | NA | NR | NA |
| (Bishop et al., 2014) | 49 (Control= 26) | NR | NR | NR | NR | NR | NR | myocardial infarction/hypertension/CHF/PVD/Pulmonary disease/IDDM/NIDDM/Gastrointestinal/Arthritis (osteo and rheumatoid)/Cancer history/Other=2/14/0/0/1/3/1/7/3/1/9 | myocardial infarction/hypertension/CHF/PVD/Pulmonary disease/IDDM/NIDDM/Gastrointestinal/Arthritis (osteo and rheumatoid)/Cancer history/Other=5/20/2/2/6/7/2/2/5/3/4 |

Note. NR: none reported; NA: not applicable.

Bishop, D., Miller, I., Weiner, D., Guilmette, T., Mukand, J., Feldmann, E., Keitner, G., Springate, B., 2014. Family Intervention: Telephone Tracking (FITT): a pilot stroke outcome study. Topics in stroke rehabilitation 21 Suppl 1, S63-74.

Cai, L.N., Shi, J.L., Chen, X.L., 2021. Effectiveness of "hospital-community-family" extended care in elderly patients with ischemic stroke. Nursing of Integrated Traditional Chinese and Western Medicine 7 (8), 136-138.

Cao, Q.R., Feng, S.W., Huang, S.Y., Li, X.Y., Lin, X.Y., Tan, Y.M., Xu, C.L., 2016. Study of home rehabilitation model and effectiveness analysis on stroke patients in rural area. Chinese Journal of Rehabilitation Theory and Practice 31 (3), 190-192.

Cao, Q.Y., Zhong, H.M., Liu, Q.E., Zhang, H.M., Liao, Y.Y., 2013. Application of group therapy in social rehabilitation training on stroke patients. China Medicine and Pharmacy 3 (11), 47-48.

Chen, L., 2021. The effect of hospital-community-family rehabilitation nursing model on the psychological state and daily living ability of patients with cerebral infarction. Heilongjiang Journal of Traditional Chinese Medicine 50 (3), 232-233.

Chen, Q., Shi, H.Q., Wu, Z.H., Zhi, B.H., Shi, M.F., 2018. The role of hospital-community-family extended rehabilitation nursing intervention in improving physical function and psychological status of stroke patients. Chronic Pathematology Journal 19 (2), 228-230.

Chen, R.X., Guan, Z.J., Lu, Y.X., Huang, K.F., Deng, L.M., Chen, S.Y., 2012. Impact of a combined hospital-community-home care model on the quality of life of depressed patients after stroke. International Medicine & Health Guidance News 18 (15), 2166-2168.

Chen, R.X., Guan, Z.J., Wu, Y.E., Fang, Y.G., Wei, Y.L., Fang, Y.H., 2012. Effects of care provided by hospital, community and family on quality of life of stroke patients. Journal of Nursing Science 27 (8), 82-84.

Chen, S., 2018. Effect of the hospital-community linkage nursing management mode on the rehabilitation and quality of life in patients with stroke. Journal of Bengbu Medical College 43 (1), 110-113.

Chen, W., Jiang, B., Zhu, H.X., Yang, Z.J., Xu, G.Z., Peng, L., Wei, J., 2019. The effect of rehabilitation of family doctor team intervention on community post-stroke depression patients. Shanghai Medical & Pharmaceutical Journal 40 (10), 56-58.

Chen, W.P., Lin, D., Lu, W., 2020. A study on the application of functional gait training in a community-based realistic environment in elderly patients in the rehabilitation period after stroke. Journal of Nursing and Rehabilitation 19 (5), 65-68.

Chi, X.Q., 2009. Community care of nine cases of hemiplegic stroke with motor function rehabilitation. Chinese Journal of Rural Medicine and Pharmacy 16 (3).

Dan, S.C., Gao, L., Ge, X.H., He, Q., 2015. Effect of Community Rehabilitation on activity of daily living in stroke patients. Heilongjiang Medicine Journal 28 (4), 887-889.

Daviet, J.C., Compagnat, M., Bonne, G., Maud, L., Bernikier, D., Salle, J.Y., 2023. Individualized home-based rehabilitation after stroke in France: a pragmatic study of a community stroke rehabilitation team. Canadian Journal of Neurological Sciences 50 (3), 405-410.

Eng, J.J., Chu, K.S., Kim, C.M., Dawson, A.S., Carswell, A., Hepburn, K.E., 2003. A community-based group exercise program for persons with chronic stroke. Medicine and Science in Sports and Exercise 35 (8), 1271-1278.

Fu, X.M., Jin, S.J., Zeng, X.L., Chen, Y., 2019. Effect of home pension system under medical-nursing combined model on quality of life in elderly patients with stroke. Hainan Medical Journal 30 (10), 1352-1355.

Gao, C.H., Huang, X.L., Zhang, W., Cai, J.H., Liu, Y.L., Wang, W., 2014. The effects of core stability training on stroke patients' motor function. Stroke and Nervous Diseases 21 (4), 207-211.

Gao, S.F., Sun, P.Y., Jiao, L.Q., 2013. Effects of different community rehabilitation models on daily living ability and neuropsychology of patients with cerebral infarction. Chinese Journal of Integrative Medicine on Cardio-/Cerebrovascular Disease 11 (7), 826-827.

He, M.L., Xie, Y.H., Wang, W.H., Li, H.S., 2019. The effect of community-based home rehabilitation care model on improving the psychological status of stroke patients. Journal of Qilu Nursing 25 (3), 105-107.

He, Y., 2015. Exploring the effectiveness of community-based rehabilitation guidance in stroke. Scientific & Technical Information of Gansu 44 (6), 116-117.

Hu, S.H., Ling, Q., Xu, J., Jiang, L.J., Lu, Y., Su, N., Hu, J.Q., Zhang, X.F., Shen, M.H., Li, R.Y., 2016. Intervention effect of community rehabilitation model in stroke patients based on regional medical association. Chinese General Practice 19 (22), 2729-2733.

Hu, X.X., Li, H., 2016. Effect of community care and family members' participation on rehabilitation of patients with cerebral infarction. Modern Clinical Nursing 15 (5), 26-30.

Huang, C.X., Zhao, S.H., 2011. The impact of process management on quality control of community care for stroke patients. Chinese General Practice 14 (35), 4028-4031.

Huang, W.L., He, Y.J., 2012. Influence of quantitative nursing intervention on rehabllitation of community stroke patients. Modern Hospitals 12 (7), 151-153.

Jiang, M.H., Qin, B., Chen, Q.G., 2010. The effect of community rehabilitation on the quality of life of homebound stroke patients with hemiplegia. Nursing Practice and Research 7 (13), 117-119.

Jiao, L.Q., Gao, S.F., Sun, P.Y., 2013. A clinical study of comprehensive community-based rehabilitation for stroke hemiparesis directed by a general hospital. Chinese Journal of Integrative Medicine on Cardio-/Cerebrovascular Disease 11 (10), 1218-1219.

Lan, Q., Ji, M.L., Chen, L.Q., 2008. A study of the effectiveness of stroke home rehabilitation team interventions for patients in the community. Shanghai Nursing 8 (4), 34-36.

Lee, D., Fischer, H., Zera, S., Robertson, R., Hammel, J., 2017. Examining a participation-focused stroke self-management intervention in a day rehabilitation setting: a quasi-experimental pilot study. Topics in Stroke Rehabilitation 24 (8), 601-607.

Li, G.Z., 2014. Effectiveness of Orem's self-care theory in the community care of stroke patients. Chinese Journal of Trauma and Disability Medicine 22 (6), 273-274.

Li, H., 2020. Hospital-community-family interface continuity of care in patients recovering from stroke. Shanxi Medical Journal 49 (13), 1748-1750.

Li, L., Li, S.W., Zhao, H., Pan, H., Zhang, L., Han, S., Zhao, L., Wu, G.S., Mao, J.F., Li, Y., 2014. Effect evaluation of the application of community rehabilitation pathway in home rehabilitation. Heilongjiang Medical Journal 38 (11), 1331-1334.

Li, L., Yue, P., Zhang, Y., 2019. The effect of hospital-community-family rehabilitation nursing model on medical compliance behavior and daily living ability of hemiplegic patients with cerebral infarction. Henan Medical Research 28 (5), 913-915.

Li, X.M., 2017. Effectiveness of community-based rehabilitation therapy in improving activities of daily living of stroke patients. Biped and Health 26 (24), 59-60.

Li, X.P., Wang, L., Lan, Y.L., Huang, W.D., Zhang, Q., 2010. Impact of community home-based rehabilitation nursing on psychological state of cerebral apoplexy patients in urban communities. Chinese Nursing Researsh 24 (3), 838-839.

Li, X.P., Wang, L., Zhang, Q., Huang, W.D., Lai, G.F., 2011. Effectiveness study of nursing by rehabilitation collaboration network among stroke family in urban community. Journal of Nurses Training 26 (9), 773-776.

Liang, N., Wang, Z.K., Zhang, Z.W., Chen, D., Lu, X.J., Song, X., Chen, R.Q., Hu, Y.Y., He, Q.C., Qin, Q.Q., 2016. Influence of community psychological intervention based on five-element theory on neural function in patients with post-stroke depression. Internal Medicine 11 (2), 174-176.

Liao, Q.H., Wang, F., Xu, W.W., Zhi, J.F., Chen, S.L., Li, J., 2019. Impact of a community-based stroke rehabilitation model on neurological rehabilitation in recovery from cerebral infarction. Zhejiang Clinical Medical Journal 21 (2), 204-206.

Liu, C.F., Zhang, J., Li, X.X., Chen, Y.R., Zhang, Z.X., Sun, X.Y., Miao, Y.Z., Wang, L.L., 2022. Effect of collaborative rehabilitation intervention on functional recovery and quality of life in elderly stroke patients. Chinese Journal of Practical Nervous Diseases 25 (2), 197-201.

Liu, H.L., Zhou, B., Zhao, Z., Yang, Y., Lv, X.Q., Wang, Y., Yu, T., 2021. Home-based telerehabilitation guidance for stroke patients. Chinese Journal of Rehabilitation Theory and Practice 27 (7), 807-811.

Liu, S.S., 2015. Take the family as the influence of self-management mode on rehabilitation of patients with cerebral apoplexy. China Health Industry 12 (8), 142-144.

Liu, Y., Liu, J.J., Jin, J.P., 2019. The efficacy of the "three-society linkage" model for community-based rehabilitation of stroke patients. Chinese Journal of Gerontology 39 (5), 1051-1053.

Lv, Q.F., 2020. Analysis of the effect of home care knowledge training in community-based elderly people recovering from stroke. Reflexology and Rehabilitation Medicine (9), 167-168.

Mao, J.B., Hu, H.J., Zhang, J.M., 2018. Influence of community family doctor as the center on rehabilitation of functional recovery of patients with stroke. Shanghai Medical & Pharmaceutical Journal 39 (8), 60-62.

Meng, F.Y., Wang, Y., 2015. Influence of continuity nursing on self-care ability of cerebral apoplexy patients in community during rehabilitation. Chinese Nursing Researsh 29 (6), 2215-2218.

Meng, Y.Q., Liu, G.J., Fan, Z.C., Qi, H., Bao, S.R.L., Zhu, R.X., 2022. Application of remote scientific rehabilitation guidance and education in hemiplegia rehabilitation. International Medicine & Health Guidance News 28 (15), 2127-2131.

Moore, S.A., Hallsworth, K., Jakovljevic, D.G., Blamire, A.M., He, J.B., Ford, G.A., Rochester, L., Trenell, M.I., 2015. Effects of community exercise therapy on metabolic, brain, physical, and cognitive function following stroke: a randomized controlled pilot trial. Neurorehabilitation and Neural Repair 29 (7), 623-635.

Park, Y.-J., Lee, C.-Y., 2016. Effects of community-based rehabilitation program on activities of daily living and cognition in elderly chronic stroke survivors. Journal of physical therapy science 28 (11), 3264-3266.

Qin, Y., Li, X.P., Wang, L., 2011. Observation on the effect of community rehabilitation nursing care for patients with post-stroke sequelae. Journal of Nursing (China) 18 (6), 63-65.

Rao, R., Ye, D., Hu, J., 2014. A study of out-of-hospital acceptance of continuity of care services for patients recovering from stroke. Chinese Journal of Rehabilitation 29 (6), 453-454.

Shi, S.X., Xu, W.W., Liu, X., Huang, J., Guo, Y., Fu, L., Jin, H., 2022. Study on the efficacy of community-led home rehabilitation model on stroke patients under the background of Internet plus. Journal of Modern Medicine & Health 38 (17), 2904-2907.

Wang, H., Wu, H., Wang, S.P., 2020. The effects of contracted services of family physicians on self-perceived burden and psychological status of stroke patients in a community. Chinese Nursing Management 20 (2), 276-281.

Wang, J.J., Xie, P., Bai, J.X., Cai, G.L., 2022. Impact of hospital-community-home continuity of care on stroke patients with non-dementia cognitive impairment. Today Nurse 29 (2), 84-87.

Wang, L., 2005. Neurologist participates in the community intervention for the functional prognosis of convalescent patients with stroke. Chinese Journal of Clinical Rehabilitation 9 (17), 4-5.

Wang, M.H., 2016. Analysis of the effect of implementing appropriate community-based rehabilitation interventions on motor function in 60 stroke patients. Chinese Journal of Trauma and Disability Medicine 24 (15), 55-56.

Wei, X.P., Yu, L.M., Hu, W., 2009. Observation of curative effects on treatment of patients with limb disability sequela after stroke in community health service. Journal of Neurology and Neurorehabilitation 6 (3), 191-193.

Wu, L., 2020. Effective application of community rehabilitation nursing in the rehabilitation management of patients with sequelae of cerebral infarction. Yi Shou Bao Dian (10), 0058-0058.

Wu, L.B., 2017. An analysis of the effects of community-based rehabilitation for stroke patients. Contemporary Medicine Symposium 15 (10), 59-60.

Wu, M.H., Zhu, C.P., Xu, X.F., Lu, A.M., Chu, H.F., 2017. Effect of home-based rehabilitation nursing on the ability of activities of daily living in the rural patients with stroke. Shanghai Medical & Pharmaceutical Journal 38 (22), 60-62.

Xia, W.M., Zhu, P., 2004. The effect of community rehabilitation on motor function of post-stroke patients. Clinical Journal of Medical Officer 32 (5), 97-98.

Xiao, X.F., 2013. Effects of community rehabilitati0n on recovery in patients with stroke and quality of life. China Modern Medicine 20 (26), 162-163.

Xu, H., Xin, Y.Y., Yin, H.X., Wang, Y.L., Wang, X.F., Tan, Y.J., 2020. Effect of hospital-community-family rehabilitation nursing model on hemiplegic patients with acute cerebral infarction. China Modern Medicine 27 (5), 239-242.

Xue, B., Gu, W.Q., Tang, Z.Y., Liu, T.L., Zhao, S., Qin, D., 2015. Study of the impact of the family rehabilitation on the life quality, anxiety and depression of the patients with the stroke under the community team mode. Shanghai Medical & Pharmaceutical Journal 36 (20), 60-62.

Xue, B., Tang, Z.Y., Liu, T.L., Zhao, S., Qin, D., Gu, W.Q., 2016. Effects of home rehabilitation in a community team model on physical function and activities of daily living in stroke patients. Shanxi Medical Journal 45 (6), 727-729.

Yang, Y., Ma, Y.H., Zhao, Z., Zhou, B., Liu, H.L., 2019. Study on the advantages and effects of applying community-based tele-rehabilitation for patients with cerebral infarction. World Latest Medicine Information 19 (24), 84-89.

Yu, J., Hu, Y., Wu, Y., Chen, W., Zhu, Y., Cui, X., Lu, W., Qi, Q., Qu, P., Shen, X., 2009. The effects of community-based rehabilitation on stroke patients in China: a single-blind, randomized controlled multicentre trial. Clinical Rehabilitation 23 (5), 408-417.

Yu, X.Y., Sang, S.H., Chi, J., Du, X., Ren, Q.H., Zhang, L., 2022. Application of information-based medical collaborative management and family cooperative management in stroke patients taking rehabilitation at home. International Medicine & Health Guidance News 28 (11), 1524-1529.

Zhang, W.F., Chen, L.N., 2018. Effects of mobile phone App based extended nursing care on the stroke patients in community. China Journal of Modern Nursing 24 (2), 190-195.

Zhang, X.C., Li, S.H., Qian, Y.F., Wang, L.Q., Yu, B., 2010. Observation on the effect of home care knowledge training for elderly people recovering from stroke in the community. Journal of Nursing (China) 17 (11), 65-67.

Zhang, X.M., Zhu, W.H., Jiang, Q.K., Gan, L.F., 2020. Efficiency of community and family-based rehabilitation strategy in the rehabilitation of post-stroke depression patients. Chinese journal of Clinical Medicine 27 (4), 657-661.

Zhang, X.Q., Wang, C., Bi, Z.Z., Gu, Y.M., Jin, L.J., Jin, R.X., Chen, L.B., Liu, Y.L., 2015. Establishment of the community stroke rehabilitation unit and evaluation of its operational result. Shanghai Medical & Pharmaceutical Journal 36 (4), 57-59.

Zhang, X.Q., Wang, C., Bi, Z.Z., Gu, Y.M., Liu, Y.L., Jin, L.J., Jin, R.X., Chen, L.B., 2015. Effect of the community-based rehabilitation unit on stroke: a prospective randomized, control study. Shanghai Medical & Pharmaceutical Journal 36 (12), 47-50.

Zhang, Y., Wang, L., Liu, Y., Gao, Y., Qian, F., Wang, Y.Q., 2022. Application effects of community elderly health service model in home health management of stroke patients. Chinese Nursing Management 22 (3), 334-338.

Zhu, J.X., Zhou, F.Y., Deng, H.D., Li, Y.L., 2014. The role of home beds in community-based rehabilitation of patients recovering from stroke. Chinese Journal for Clinicians 42 (6), 41-42.
