# Supplementary material for: Community-based rehabilitation services implemented by multidisciplinary teams among adults with stroke: a scoping review with a focus on Chinese experience
Source: BMC Public Health. 2024 Mar 7;24:740. doi: 10.1186/s12889-024-18218-1 (PMC10921794; doi:10.1186/s12889-024-18218-1)
Supplement: Supplementary file 7 — Supplementary Material 7 [file 12889_2024_18218_MOESM7_ESM.docx]

Table S6 Outcome measures for each included study (N=74)

| **Author** | **Functional outcomes  (Tools)** | **Body composition  (Tools)** | **Psychosocial outcomes  (Tools)** | **Cardiovascular health  (Tools)** | **Dietary outcomes  (Tools)** |
| --- | --- | --- | --- | --- | --- |
| (Wang et al., 2022) | Neurological deficits (NIHSS**)  ADLs (ADL**) | NA | Cognitive function (MoCA, MMSE**) | NA | NA |
| (Zhang and Chen, 2018) | Motor function (FMA*) | Total cholesterol**, HDL**, homocysteine** | Stroke self-efficacy questionnaire**, quality of life (SF-36- dimensions*) | NA | NA |
| (Lan et al., 2008) | Motor function (STREAM**)  ADLs (FIM**) | NA | NA | NA | NA |
| (Chen, 2021) | ADLs (BI**) | NA | Depression (PHQ-9**), anxiety (GAD-7**) | NA | NA |
| (Zhang et al., 2015) | Neurological deficits (NIHSS*)  ADLs (MBI*) | NA | NA | NA | NA |
| (Jiang et al., 2010) | ADLs (BI**) | NA | Depression (SDS**), anxiety (SAS**) | NA | NA |
| (Chen et al., 2012) | NA | NA | Depression (HRSD**), quality of life (SS-QOL**) | NA | NA |
| (Chen, 2018) | ADLs (BI**), Neurological deficits (NIHSS**), motor function (FMA**) | NA | Quality of life (GQOLI-74**) | NA | NA |
| **Author** | **Functional outcomes  (Tools)** | **Body composition  (Tools)** | **Psychosocial outcomes  (Tools)** | **Cardiovascular health  (Tools)** | **Dietary outcomes  (Tools)** |
| (Cai et al., 2021) | Neurological deficits (NIHSS**), ADLs (MBI*), complications* | NA | Depression (HAMD*), anxiety (HAMA*), quality of life (SS-QOL**), rehabilitation compliance** | NA | NA |
| (Fu et al., 2019) | ADLs (BI*) | NA | Quality of life (SS-QOL**) | NA | NA |
| (Chi, 2009) | ADLs (BI^), myodynamia (Lovett scale^) | NA | NA | NA | NA |
| (Wu et al., 2017) | ADLs (BI*) | NA | NA | NA | NA |
| (Chen et al., 2012) | Neurological deficits (NIHSS**) | NA | Quality of life (SS-QOL**) | NA | NA |
| (Zhang et al., 2020) | ADLs (FCA*) | NA | Depression (HRSD**), quality of life (WHOQOL-BREF^#^-physical dimension*) | NA | NA |
| (Dan et al., 2015) | ADLs (BI*) | NA | Cognitive function (MMSE*) | NA | NA |
| (He, 2015) | Neurological deficits (NIHSS*)  ADLs (BI*) | NA | NA | NA | NA |
| (Huang and He, 2012) | Motor function (FMA**), ADLs (ADL**) | NA | Depression (SDS**), anxiety (SAS**) | NA | NA |
| (Rao et al., 2014) | NA | NA | Stroke rehabilitation knowledge*, self-care agency (ESCA*) | NA | NA |
| **Author** | **Functional outcomes  (Tools)** | **Body composition  (Tools)** | **Psychosocial outcomes  (Tools)** | **Cardiovascular health  (Tools)** | **Dietary outcomes  (Tools)** |
| (Wei et al., 2009) | Myodynamia (MMT^&^), ADLs (ADL**) | NA | NA | NA | NA |
| (Gao et al., 2013) | ADLs (BI*) | NA | Depression (HAMD*), anxiety (HAMA*) | NA | NA |
| (Shi et al., 2022) | Motor function (FMA*), ADLs (MBI*), | NA | anxiety (SAS*), quality of life (QLI*) | NA | NA |
| (Liu et al., 2019) | NA | NA | Depression (SDS*), quality of life (SS-QOL*) | NA | NA |
| (Li et al., 2011) | Motor function (FMA**), ADLs (BI**) | NA | Depression (DIS**), quality of life (SS-QOL^#^- * or ** except for visual acuity dimension), family function (APGAR^&^), social support (SSRS*) | NA | NA |
| (Hu and Li, 2016) | ADLs (MBI**, FCA**) | NA | NA | NA | NA |
| (Li, 2014) | Motor function (FMA**), ADLs (BI**) | NA | NA | NA | NA |
| (Li, 2020) | Motor function (FMA*), ADLs (BI*) | NA | NA | NA | NA |
| (He et al., 2019) | Motor function (FMA*) | NA | Depression (DSI*) | NA | NA |
| (Hu et al., 2016) | Motor function (FMA*), ADLs (BI*) | NA | NA | NA | NA |
| (Zhang et al., 2022) | ADLs (MBI**) | NA | quality of life (SF-36*) | NA | NA |
| **Author** | **Functional outcomes  (Tools)** | **Body composition  (Tools)** | **Psychosocial outcomes  (Tools)** | **Cardiovascular health  (Tools)** | **Dietary outcomes  (Tools)** |
| (Chen et al., 2019) | ADLs (ADL^$^-12 weeks*, 24 weeks**, 36 weeks**) | NA | Depression (HAMD**, SDS^$^-12 weeks*, 24 weeks**, 36 weeks**) | NA | NA |
| (Wu, 2017) | ADLs (BI*) | NA | Quality of life (self-made questionnaire*) | NA | NA |
| (Wu, 2020) | ADLs (BI*) | NA | Quality of life (EORTC QLQ-C30*) | NA | NA |
| (Xiao, 2013) | ADLs (BI*) | NA | Cognitive function (MMSE*) | NA | NA |
| (Cao et al., 2016) | ADLs (MBI*) | NA | Depression (SDS*), anxiety (SAS*), quality of life (QLI*) | NA | NA |
| (Liu et al., 2021) | Motor function (FMA**), ADLs (MBI**) | NA | NA | NA | NA |
| (Zhang et al., 2010) | ADLs (BI*) | NA | Depression (HAMD**), quality of life (QLI*), stroke knowledge** | NA | NA |
| (Mao et al., 2018) | Neurological deficits (NIHSS*), motor function (FMA*), ADLs (BI*) | NA | NA | NA | NA |
| (Yu et al., 2022) | ADLs (BI**) | NA | Rehabilitation compliance**, degree of satisfaction* | NA | NA |
| **Author** | **Functional outcomes  (Tools)** | **Body composition  (Tools)** | **Psychosocial outcomes  (Tools)** | **Cardiovascular health  (Tools)** | **Dietary outcomes  (Tools)** |
| (Li et al., 2010) | ADLs (FIM*) | NA | Depression (DSI*) | NA | NA |
| (Li et al., 2019) | ADLs (BI**) | NA | Rehabilitation compliance** | NA | NA |
| (Huang and Zhao, 2011) | ADLs (BI**) | NA | Stroke knowledge**, degree of satisfaction** | NA | NA |
| (Xue et al., 2016) | Motor function (FMA*), ADLs (BI*) | NA | NA | NA | NA |
| (Meng et al., 2022) | Motor function (FMA**), ADLs (FIM**) | NA | NA | NA | NA |
| (Xia and Zhu, 2004) | Motor function (MAS**), ADLs (MBI**) | NA | NA | NA | NA |
| (Xue et al., 2016) | Motor function (FMA*), ADLs (MBI*) | NA | NA | NA | NA |
| (Yang et al., 2019) | Motor function (FMA*), ADLs (BI*) | NA | NA | NA | NA |
| (Liao et al., 2019) | Motor function (FMA*), ADLs (BI*) | NA | Quality of life (SS-QOL**) | cerebral hemodynamics (TCD): Vp*, Vm*, DVp*, DVm*, CVR*, BHI* | NA |
| (Wang, 2005) | Neurological deficits (ESS*), the number and incidence of recurrent stroke within 3 years*, ADLs (MBI*) | Blood glucose**, blood lipid**, fibrinogen* | Dementia rating (CDR*) | Blood pressure** | NA |
| **Author** | **Functional outcomes  (Tools)** | **Body composition  (Tools)** | **Psychosocial outcomes  (Tools)** | **Cardiovascular health  (Tools)** | **Dietary outcomes  (Tools)** |
| (Li, 2017) | Recovery (ADL*), ADLs (ADL*) | NA | NA | NA | NA |
| (Li et al., 2014) | Motor function (BRS**, FMA**), ADLs (BI^#^ Significant except for no significance in bowel control*) | NA | Self-care ability**, quality of life (WHOQOL-BREF^) | NA | NA |
| (Zhu et al., 2014) | Motor function (FMA*), static balance and fall risk (BBS*), ADLs (MBI*, FIM*) | NA | NA | NA | NA |
| (Cao et al., 2013) | Static balance and fall risk (BBS**), ADLs (MBI**) | NA | NA | NA | NA |
| (Liu, 2015) | Motor function (FMA**) | NA | Quality of life (SIS^) | NA | NA |
| (Zhang et al., 2015) | ADLs (BI*) | NA | Degree of satisfaction* | NA | NA |
| (Chen et al., 2020) | Falls efficacy (MFES*), incidence of falls* | NA | Fear of falling*, degree of satisfaction* | NA | NA |
| (Lv, 2020) | ADLs (BI**) | NA | Depression (SDS**), quality of life (QLI**), stroke knowledge** | NA | NA |
| (Xue et al., 2015) | NA | NA | Depression (HAHD**), anxiety (HAMA**), quality of life (QLI**) | NA | NA |
| **Author** | **Functional outcomes  (Tools)** | **Body composition  (Tools)** | **Psychosocial outcomes  (Tools)** | **Cardiovascular health  (Tools)** | **Dietary outcomes  (Tools)** |
| (Chen et al., 2018) | Motor function (FMA**), ADLs (BI**) | NA | Depression (HAMD**) | NA | NA |
| (Jiao et al., 2013) | Motor function (FMA*), balance function (FIM-B*), ADLs (MBI*) | NA | NA | NA | NA |
| (Liang et al., 2016) | Neurological deficits (NIHSS*), ADLs (BI**) | NA | Depression (HAMD**), cognitive function (MMSE**) | NA | NA |
| (Xu et al., 2020) | Neurological deficits (NIHSS**), ADLs (BI**) | NA | Depression (SDS**), anxiety (SAS**) | NA | NA |
| (Qin et al., 2011) | Motor function (FMA*), ADLs (MBI**) | NA | NA | NA | NA |
| (Gao et al., 2014) | Trunk control (TCT*), motor function (FMA*), static balance and fall risk (BBS*), walking ability (FAC*), gait (foot print analysis*), ADLs (MBI*) | NA | NA | NA | NA |
| (Liu et al., 2022) | NA | Urinary incontinence effect* | Quality of life (I-QOL*) | NA | NA |
| (Wang, 2016) | Motor function (FMA*), ADLs (BI*) | NA | NA | NA | NA |
| (Wang et al., 2020) | Neurological deficits (NIHSS^), ADLs (mRS**, MBI^$^- 3 months**, 6 months^&^) | NA | Depression (SDS), anxiety (SAS**), self-perceived burden (SPBS**) | NA | NA |
| **Author** | **Functional outcomes  (Tools)** | **Body composition  (Tools)** | **Psychosocial outcomes  (Tools)** | **Cardiovascular health  (Tools)** | **Dietary outcomes  (Tools)** |
| (Meng and Wang, 2015) | NA | NA | Continuity of care (NCQ^#^-1 dimension is meaningless, the remaining 3 are meaningful**), self-care agency (ESCA**) | NA | NA |
| (Moore et al., 2015) | Physical performance: 6-minute walk test**, 10-m walk test**, and static balance and fall risk (BBS**) | Body mass index^&^, lipid Profile (HDL-C**), blood glucose^&^, insulin sensitivity (HOMA^&^) | Cognitive function (ACE-R*); quality of life (SIS, stroke recovery and mood*) | Cerebral tissue atrophy^&^, blood flow*, and regional metabolism (MRI^&^),  a maximal progressive recumbent bicycle exercise test**, blood pressure (DBP*) | NA |
| (Yu et al., 2009) | Clinical Neurological Function Deficit Scale (0-2m**, 2-5m**) | NA | NA | NA | NA |
| (Daviet et al., 2023) | ADLs (FAI**, MBI**) | NA | Caregiver burden (Mini-Zarit**); quality of life (the Euroqol**) | NA | NA |
| (Eng et al., 2003) | The 12-minute walk test*, static balance and fall risk (BBS*), gait Speed* | NA | Recreation and social participation (RNLI^&^), satisfaction (COPM*) | NA | NA |
| (Lee et al., 2017) | NA | NA | Quality of life (SIS, Mobility*, Perceived recovery*), recreation and social participation (RNLI^&^), self-efficacy (PS-SES^&^) | NA | NA |
| **Author** | **Functional outcomes  (Tools)** | **Body composition  (Tools)** | **Psychosocial outcomes  (Tools)** | **Cardiovascular health  (Tools)** | **Dietary outcomes  (Tools)** |
| (Park and Lee, 2016) | ADLs (MBI*) | NA | Cognitive function (MMSE*), | NA | NA |
| (Bishop et al., 2014) | ADLs (FAI:3m-caregiver*, FIM^&^) | NA | Depression (GDS^&^), family function (FAD:6m*, PCS1*, PCS2:3m-stroke patients*) | NA | NA |

Note. **P*<0.05, ***P*<0.01; ^&^*P*＞0.05, ^#^ *P*: Only some dimensions are significant, ^$^ *P*: Difference in significance at different follow-up points, ****P*<0.05 or <0.01, ^ *P*: No statistical comparisons were made; NA: not applicable.

Bishop, D., Miller, I., Weiner, D., Guilmette, T., Mukand, J., Feldmann, E., Keitner, G., Springate, B., 2014. Family Intervention: Telephone Tracking (FITT): a pilot stroke outcome study. Topics in stroke rehabilitation 21 Suppl 1, S63-74.

Cai, L.N., Shi, J.L., Chen, X.L., 2021. Effectiveness of "hospital-community-family" extended care in elderly patients with ischemic stroke. Nursing of Integrated Traditional Chinese and Western Medicine 7 (8), 136-138.

Cao, Q.R., Feng, S.W., Huang, S.Y., Li, X.Y., Lin, X.Y., Tan, Y.M., Xu, C.L., 2016. Study of home rehabilitation model and effectiveness analysis on stroke patients in rural area. Chinese Journal of Rehabilitation Theory and Practice 31 (3), 190-192.

Cao, Q.Y., Zhong, H.M., Liu, Q.E., Zhang, H.M., Liao, Y.Y., 2013. Application of group therapy in social rehabilitation training on stroke patients. China Medicine and Pharmacy 3 (11), 47-48.

Chen, L., 2021. The effect of hospital-community-family rehabilitation nursing model on the psychological state and daily living ability of patients with cerebral infarction. Heilongjiang Journal of Traditional Chinese Medicine 50 (3), 232-233.

Chen, Q., Shi, H.Q., Wu, Z.H., Zhi, B.H., Shi, M.F., 2018. The role of hospital-community-family extended rehabilitation nursing intervention in improving physical function and psychological status of stroke patients. Chronic Pathematology Journal 19 (2), 228-230.

Chen, R.X., Guan, Z.J., Lu, Y.X., Huang, K.F., Deng, L.M., Chen, S.Y., 2012. Impact of a combined hospital-community-home care model on the quality of life of depressed patients after stroke. International Medicine & Health Guidance News 18 (15), 2166-2168.

Chen, R.X., Guan, Z.J., Wu, Y.E., Fang, Y.G., Wei, Y.L., Fang, Y.H., 2012. Effects of care provided by hospital, community and family on quality of life of stroke patients. Journal of Nursing Science 27 (8), 82-84.

Chen, S., 2018. Effect of the hospital-community linkage nursing management mode on the rehabilitation and quality of life in patients with stroke. Journal of Bengbu Medical College 43 (1), 110-113.

Chen, W., Jiang, B., Zhu, H.X., Yang, Z.J., Xu, G.Z., Peng, L., Wei, J., 2019. The effect of rehabilitation of family doctor team intervention on community post-stroke depression patients. Shanghai Medical & Pharmaceutical Journal 40 (10), 56-58.

Chen, W.P., Lin, D., Lu, W., 2020. A study on the application of functional gait training in a community-based realistic environment in elderly patients in the rehabilitation period after stroke. Journal of Nursing and Rehabilitation 19 (5), 65-68.

Chi, X.Q., 2009. Community care of nine cases of hemiplegic stroke with motor function rehabilitation. Chinese Journal of Rural Medicine and Pharmacy 16 (3).

Dan, S.C., Gao, L., Ge, X.H., He, Q., 2015. Effect of Community Rehabilitation on activity of daily living in stroke patients. Heilongjiang Medicine Journal 28 (4), 887-889.

Daviet, J.C., Compagnat, M., Bonne, G., Maud, L., Bernikier, D., Salle, J.Y., 2023. Individualized home-based rehabilitation after stroke in France: a pragmatic study of a community stroke rehabilitation team. Canadian Journal of Neurological Sciences 50 (3), 405-410.

Eng, J.J., Chu, K.S., Kim, C.M., Dawson, A.S., Carswell, A., Hepburn, K.E., 2003. A community-based group exercise program for persons with chronic stroke. Medicine and Science in Sports and Exercise 35 (8), 1271-1278.

Fu, X.M., Jin, S.J., Zeng, X.L., Chen, Y., 2019. Effect of home pension system under medical-nursing combined model on quality of life in elderly patients with stroke. Hainan Medical Journal 30 (10), 1352-1355.

Gao, C.H., Huang, X.L., Zhang, W., Cai, J.H., Liu, Y.L., Wang, W., 2014. The effects of core stability training on stroke patients' motor function. Stroke and Nervous Diseases 21 (4), 207-211.

Gao, S.F., Sun, P.Y., Jiao, L.Q., 2013. Effects of different community rehabilitation models on daily living ability and neuropsychology of patients with cerebral infarction. Chinese Journal of Integrative Medicine on Cardio-/Cerebrovascular Disease 11 (7), 826-827.

He, M.L., Xie, Y.H., Wang, W.H., Li, H.S., 2019. The effect of community-based home rehabilitation care model on improving the psychological status of stroke patients. Journal of Qilu Nursing 25 (3), 105-107.

He, Y., 2015. Exploring the effectiveness of community-based rehabilitation guidance in stroke. Scientific & Technical Information of Gansu 44 (6), 116-117.

Hu, S.H., Ling, Q., Xu, J., Jiang, L.J., Lu, Y., Su, N., Hu, J.Q., Zhang, X.F., Shen, M.H., Li, R.Y., 2016. Intervention effect of community rehabilitation model in stroke patients based on regional medical association. Chinese General Practice 19 (22), 2729-2733.

Hu, X.X., Li, H., 2016. Effect of community care and family members' participation on rehabilitation of patients with cerebral infarction. Modern Clinical Nursing 15 (5), 26-30.

Huang, C.X., Zhao, S.H., 2011. The impact of process management on quality control of community care for stroke patients. Chinese General Practice 14 (35), 4028-4031.

Huang, W.L., He, Y.J., 2012. Influence of quantitative nursing intervention on rehabllitation of community stroke patients. Modern Hospitals 12 (7), 151-153.

Jiang, M.H., Qin, B., Chen, Q.G., 2010. The effect of community rehabilitation on the quality of life of homebound stroke patients with hemiplegia. Nursing Practice and Research 7 (13), 117-119.

Jiao, L.Q., Gao, S.F., Sun, P.Y., 2013. A clinical study of comprehensive community-based rehabilitation for stroke hemiparesis directed by a general hospital. Chinese Journal of Integrative Medicine on Cardio-/Cerebrovascular Disease 11 (10), 1218-1219.

Lan, Q., Ji, M.L., Chen, L.Q., 2008. A study of the effectiveness of stroke home rehabilitation team interventions for patients in the community. Shanghai Nursing 8 (4), 34-36.

Lee, D., Fischer, H., Zera, S., Robertson, R., Hammel, J., 2017. Examining a participation-focused stroke self-management intervention in a day rehabilitation setting: a quasi-experimental pilot study. Topics in Stroke Rehabilitation 24 (8), 601-607.

Li, G.Z., 2014. Effectiveness of Orem's self-care theory in the community care of stroke patients. Chinese Journal of Trauma and Disability Medicine 22 (6), 273-274.

Li, H., 2020. Hospital-community-family interface continuity of care in patients recovering from stroke. Shanxi Medical Journal 49 (13), 1748-1750.

Li, L., Li, S.W., Zhao, H., Pan, H., Zhang, L., Han, S., Zhao, L., Wu, G.S., Mao, J.F., Li, Y., 2014. Effect evaluation of the application of community rehabilitation pathway in home rehabilitation. Heilongjiang Medical Journal 38 (11), 1331-1334.

Li, L., Yue, P., Zhang, Y., 2019. The effect of hospital-community-family rehabilitation nursing model on medical compliance behavior and daily living ability of hemiplegic patients with cerebral infarction. Henan Medical Research 28 (5), 913-915.

Li, X.M., 2017. Effectiveness of community-based rehabilitation therapy in improving activities of daily living of stroke patients. Biped and Health 26 (24), 59-60.

Li, X.P., Wang, L., Lan, Y.L., Huang, W.D., Zhang, Q., 2010. Impact of community home-based rehabilitation nursing on psychological state of cerebral apoplexy patients in urban communities. Chinese Nursing Researsh 24 (3), 838-839.

Li, X.P., Wang, L., Zhang, Q., Huang, W.D., Lai, G.F., 2011. Effectiveness study of nursing by rehabilitation collaboration network among stroke family in urban community. Journal of Nurses Training 26 (9), 773-776.

Liang, N., Wang, Z.K., Zhang, Z.W., Chen, D., Lu, X.J., Song, X., Chen, R.Q., Hu, Y.Y., He, Q.C., Qin, Q.Q., 2016. Influence of community psychological intervention based on five-element theory on neural function in patients with post-stroke depression. Internal Medicine 11 (2), 174-176.

Liao, Q.H., Wang, F., Xu, W.W., Zhi, J.F., Chen, S.L., Li, J., 2019. Impact of a community-based stroke rehabilitation model on neurological rehabilitation in recovery from cerebral infarction. Zhejiang Clinical Medical Journal 21 (2), 204-206.

Liu, C.F., Zhang, J., Li, X.X., Chen, Y.R., Zhang, Z.X., Sun, X.Y., Miao, Y.Z., Wang, L.L., 2022. Effect of collaborative rehabilitation intervention on functional recovery and quality of life in elderly stroke patients. Chinese Journal of Practical Nervous Diseases 25 (2), 197-201.

Liu, H.L., Zhou, B., Zhao, Z., Yang, Y., Lv, X.Q., Wang, Y., Yu, T., 2021. Home-based telerehabilitation guidance for stroke patients. Chinese Journal of Rehabilitation Theory and Practice 27 (7), 807-811.

Liu, S.S., 2015. Take the family as the influence of self-management mode on rehabilitation of patients with cerebral apoplexy. China Health Industry 12 (8), 142-144.

Liu, Y., Liu, J.J., Jin, J.P., 2019. The efficacy of the "three-society linkage" model for community-based rehabilitation of stroke patients. Chinese Journal of Gerontology 39 (5), 1051-1053.

Lv, Q.F., 2020. Analysis of the effect of home care knowledge training in community-based elderly people recovering from stroke. Reflexology and Rehabilitation Medicine (9), 167-168.

Mao, J.B., Hu, H.J., Zhang, J.M., 2018. Influence of community family doctor as the center on rehabilitation of functional recovery of patients with stroke. Shanghai Medical & Pharmaceutical Journal 39 (8), 60-62.

Meng, F.Y., Wang, Y., 2015. Influence of continuity nursing on self-care ability of cerebral apoplexy patients in community during rehabilitation. Chinese Nursing Researsh 29 (6), 2215-2218.

Meng, Y.Q., Liu, G.J., Fan, Z.C., Qi, H., Bao, S.R.L., Zhu, R.X., 2022. Application of remote scientific rehabilitation guidance and education in hemiplegia rehabilitation. International Medicine & Health Guidance News 28 (15), 2127-2131.

Moore, S.A., Hallsworth, K., Jakovljevic, D.G., Blamire, A.M., He, J.B., Ford, G.A., Rochester, L., Trenell, M.I., 2015. Effects of community exercise therapy on metabolic, brain, physical, and cognitive function following stroke: a randomized controlled pilot trial. Neurorehabilitation and Neural Repair 29 (7), 623-635.

Park, Y.-J., Lee, C.-Y., 2016. Effects of community-based rehabilitation program on activities of daily living and cognition in elderly chronic stroke survivors. Journal of physical therapy science 28 (11), 3264-3266.

Qin, Y., Li, X.P., Wang, L., 2011. Observation on the effect of community rehabilitation nursing care for patients with post-stroke sequelae. Journal of Nursing (China) 18 (6), 63-65.

Rao, R., Ye, D., Hu, J., 2014. A study of out-of-hospital acceptance of continuity of care services for patients recovering from stroke. Chinese Journal of Rehabilitation 29 (6), 453-454.

Shi, S.X., Xu, W.W., Liu, X., Huang, J., Guo, Y., Fu, L., Jin, H., 2022. Study on the efficacy of community-led home rehabilitation model on stroke patients under the background of Internet plus. Journal of Modern Medicine & Health 38 (17), 2904-2907.

Wang, H., Wu, H., Wang, S.P., 2020. The effects of contracted services of family physicians on self-perceived burden and psychological status of stroke patients in a community. Chinese Nursing Management 20 (2), 276-281.

Wang, J.J., Xie, P., Bai, J.X., Cai, G.L., 2022. Impact of hospital-community-home continuity of care on stroke patients with non-dementia cognitive impairment. Today Nurse 29 (2), 84-87.

Wang, L., 2005. Neurologist participates in the community intervention for the functional prognosis of convalescent patients with stroke. Chinese Journal of Clinical Rehabilitation 9 (17), 4-5.

Wang, M.H., 2016. Analysis of the effect of implementing appropriate community-based rehabilitation interventions on motor function in 60 stroke patients. Chinese Journal of Trauma and Disability Medicine 24 (15), 55-56.

Wei, X.P., Yu, L.M., Hu, W., 2009. Observation of curative effects on treatment of patients with limb disability sequela after stroke in community health service. Journal of Neurology and Neurorehabilitation 6 (3), 191-193.

Wu, L., 2020. Effective application of community rehabilitation nursing in the rehabilitation management of patients with sequelae of cerebral infarction. Yi Shou Bao Dian (10), 0058-0058.

Wu, L.B., 2017. An analysis of the effects of community-based rehabilitation for stroke patients. Contemporary Medicine Symposium 15 (10), 59-60.

Wu, M.H., Zhu, C.P., Xu, X.F., Lu, A.M., Chu, H.F., 2017. Effect of home-based rehabilitation nursing on the ability of activities of daily living in the rural patients with stroke. Shanghai Medical & Pharmaceutical Journal 38 (22), 60-62.

Xia, W.M., Zhu, P., 2004. The effect of community rehabilitation on motor function of post-stroke patients. Clinical Journal of Medical Officer 32 (5), 97-98.

Xiao, X.F., 2013. Effects of community rehabilitati0n on recovery in patients with stroke and quality of life. China Modern Medicine 20 (26), 162-163.

Xu, H., Xin, Y.Y., Yin, H.X., Wang, Y.L., Wang, X.F., Tan, Y.J., 2020. Effect of hospital-community-family rehabilitation nursing model on hemiplegic patients with acute cerebral infarction. China Modern Medicine 27 (5), 239-242.

Xue, B., Gu, W.Q., Tang, Z.Y., Liu, T.L., Zhao, S., Qin, D., 2015. Study of the impact of the family rehabilitation on the life quality, anxiety and depression of the patients with the stroke under the community team mode. Shanghai Medical & Pharmaceutical Journal 36 (20), 60-62.

Xue, B., Tang, Z.Y., Liu, T.L., Zhao, S., Qin, D., Gu, W.Q., 2016. Effects of home rehabilitation in a community team model on physical function and activities of daily living in stroke patients. Shanxi Medical Journal 45 (6), 727-729.

Yang, Y., Ma, Y.H., Zhao, Z., Zhou, B., Liu, H.L., 2019. Study on the advantages and effects of applying community-based tele-rehabilitation for patients with cerebral infarction. World Latest Medicine Information 19 (24), 84-89.

Yu, J., Hu, Y., Wu, Y., Chen, W., Zhu, Y., Cui, X., Lu, W., Qi, Q., Qu, P., Shen, X., 2009. The effects of community-based rehabilitation on stroke patients in China: a single-blind, randomized controlled multicentre trial. Clinical Rehabilitation 23 (5), 408-417.

Yu, X.Y., Sang, S.H., Chi, J., Du, X., Ren, Q.H., Zhang, L., 2022. Application of information-based medical collaborative management and family cooperative management in stroke patients taking rehabilitation at home. International Medicine & Health Guidance News 28 (11), 1524-1529.

Zhang, W.F., Chen, L.N., 2018. Effects of mobile phone App based extended nursing care on the stroke patients in community. China Journal of Modern Nursing 24 (2), 190-195.

Zhang, X.C., Li, S.H., Qian, Y.F., Wang, L.Q., Yu, B., 2010. Observation on the effect of home care knowledge training for elderly people recovering from stroke in the community. Journal of Nursing (China) 17 (11), 65-67.

Zhang, X.M., Zhu, W.H., Jiang, Q.K., Gan, L.F., 2020. Efficiency of community and family-based rehabilitation strategy in the rehabilitation of post-stroke depression patients. Chinese journal of Clinical Medicine 27 (4), 657-661.

Zhang, X.Q., Wang, C., Bi, Z.Z., Gu, Y.M., Jin, L.J., Jin, R.X., Chen, L.B., Liu, Y.L., 2015. Establishment of the community stroke rehabilitation unit and evaluation of its operational result. Shanghai Medical & Pharmaceutical Journal 36 (4), 57-59.

Zhang, X.Q., Wang, C., Bi, Z.Z., Gu, Y.M., Liu, Y.L., Jin, L.J., Jin, R.X., Chen, L.B., 2015. Effect of the community-based rehabilitation unit on stroke: a prospective randomized, control study. Shanghai Medical & Pharmaceutical Journal 36 (12), 47-50.

Zhang, Y., Wang, L., Liu, Y., Gao, Y., Qian, F., Wang, Y.Q., 2022. Application effects of community elderly health service model in home health management of stroke patients. Chinese Nursing Management 22 (3), 334-338.

Zhu, J.X., Zhou, F.Y., Deng, H.D., Li, Y.L., 2014. The role of home beds in community-based rehabilitation of patients recovering from stroke. Chinese Journal for Clinicians 42 (6), 41-42.
